# Genome-wide discovery of the daily transcriptome, DNA regulatory elements and transcription factor occupancy in the monarch butterfly brain

**DOI:** 10.1371/journal.pgen.1008265

**Published:** 2019-07-23

**Authors:** Aldrin B. Lugena, Ying Zhang, Jerome S. Menet, Christine Merlin

**Affiliations:** Department of Biology and Center for Biological Clocks Research, Texas A&M University, College Station, Texas, United States of America; Charité - Universitätsmedizin Berlin, GERMANY

## Abstract

The Eastern North American monarch butterfly, *Danaus plexippus*, is famous for its spectacular seasonal long-distance migration. In recent years, it has also emerged as a novel system to study how animal circadian clocks keep track of time and regulate ecologically relevant daily rhythmic activities and seasonal behavioral outputs. However, unlike in *Drosophila* and the mouse, little work has been undertaken in the monarch to identify rhythmic genes at the genome-wide level and elucidate the regulation of their diurnal expression. Here, we used RNA-sequencing and Assay for Transposase-Accessible Chromatin (ATAC)-sequencing to profile the diurnal transcriptome, open chromatin regions, and transcription factor (TF) footprints in the brain of wild-type monarchs and of monarchs with impaired clock function, including *Cryptochrome 2* (*Cry2*), *Clock* (*Clk*), and *Cycle-like* loss-of-function mutants. We identified 217 rhythmically expressed genes in the monarch brain; many of them were involved in the regulation of biological processes key to brain function, such as glucose metabolism and neurotransmission. Surprisingly, we found no significant time-of-day and genotype-dependent changes in chromatin accessibility in the brain. Instead, we found the existence of a temporal regulation of TF occupancy within open chromatin regions in the vicinity of rhythmic genes in the brains of wild-type monarchs, which is disrupted in clock deficient mutants. Together, this work identifies for the first time the rhythmic genes and modes of regulation by which diurnal transcription rhythms are regulated in the monarch brain. It also illustrates the power of ATAC-sequencing to profile genome-wide regulatory elements and TF binding in a non-model organism for which TF-specific antibodies are not yet available.

## Author summary

With a rich biology that includes a clock-regulated migratory behavior and a circadian clock possessing mammalian clock orthologues, the monarch butterfly is an unconventional system with broad appeal to study circadian and seasonal rhythms. While clockwork mechanisms and rhythmic behavioral outputs have been studied in this species, the rhythmic genes that regulate rhythmic daily and seasonal activities remain largely unknown. Likewise, the mechanisms regulating rhythmic gene expression have not been explored in the monarch. Here, we applied genome-wide sequencing approaches to identify genes with rhythmic diurnal expression in the monarch brain, revealing the coordination of key pathways for brain function. We also identified the monarch brain open chromatin regions and provide evidence that regulation of rhythmic gene expression does not occur through temporal regulation of chromatin opening but rather by the time-of-day dependent binding of transcription factors in *cis*-regulatory elements. Together, our data extend our knowledge of the molecular rhythmic pathways, which may prove important in understanding the mechanisms underlying the daily and seasonal biology of the migratory monarch butterflies.

## Introduction

Organisms have evolved daily and seasonal rhythms in behavior, physiology and metabolism that are driven by biological clocks to adapt to a temporally dynamic environment. The Eastern North American migratory monarch butterfly, *Danaus plexippus*, has emerged as a new system to study circadian clocks and their role in regulating daily and seasonal biology [1,2]. The sequencing of its genome [3], along with the development of CRISPR/Cas9-mediated reverse-genetics to generate monarch clock gene knockouts [4–6], have unlocked the potential of the monarch for studying animal clockwork mechanisms. Yet, in contrast to conventional model organisms like the fruit fly and the mouse, the identity of the genes expressed rhythmically over the course of the day and their *cis*-regulatory regions in the brain, the anatomical site driving daily rhythms in locomotor activity, remain unknown in the monarch system.

Migratory monarchs are famous for their iconic seasonal long-distance migration and the use of a sun compass that allows both fall migrants and spring remigrants to navigate to their respective destinations [7–13]. Circadian clocks are part of the monarch navigational toolkit as they time-compensate the sun compass output in the brain, enabling migrants and remigrants to maintain a fixed flight direction throughout the day [7–9,11,12]. These clocks may also contribute to the seasonal induction of the migratory physiology and behavior and the timing of their migration [2]. The molecular oscillator that drives monarch circadian rhythms has been deciphered and relies, similar to other eukaryotes, on a transcriptional-translational core feedback loop running with a period of ~24 hours. Interestingly, the monarch possesses a hybrid clock that contains a mammalian-like core feedback loop and a *Drosophila*-like entrainment pathway [14]. Heterodimers of the transcription factors CLOCK(CLK):BMAL1 drive the rhythmic transcription of the *cryptochrome 2* (*cry2*), *period* (*per*), and *timeless* (*tim*) genes [14,15]. Upon translation, CRY2, PER, and TIM form complexes that translocate back into the nucleus where CRY2, the orthologue of the mammalian CRY1 transcriptional repressor, inhibits CLK:BMAL1-mediated transcription [5,6,14,15]. Photic entrainment of the clock is similar to that found in the fruit fly, occurring through the circadian blue-light photoreceptor CRYPTOCHROME 1 (CRY1) and the CRY1-TIM pathway [14,16–18].

The molecular circadian clock does not just regulate the expression of core clock components. It also drives the rhythmic expression of thousands of transcripts in a tissue-specific manner such that physiological functions are tuned to optimally perform at the most appropriate time of the day. The genes rhythmically expressed in light:dark or constant dark conditions and the transcription factors (TFs) that regulate their rhythmic expression have been identified in different tissues of several organisms from insects to mammals [19–29]. However, their identity remains unknown in the monarch.

Given the potential of the monarch as a new genetic model to study how circadian clocks regulate daily and seasonal physiological and behavioral outputs, we aimed in this study at 1) identifying genes expressed rhythmically and under the control of circadian core clock components over the 24-hour light:dark cycle in the monarch brain, 2) identifying the *cis*-regulatory elements that contribute to rhythmic gene expression, and 3) characterizing the effect of clock disruption on *cis*-regulatory element accessibility and on the putative TFs that bind to them.

## Results

### Profiling of the diurnal transcriptome identifies rhythmic expression of core clock genes and clock output genes in the monarch butterfly brain

**Fig 1:**
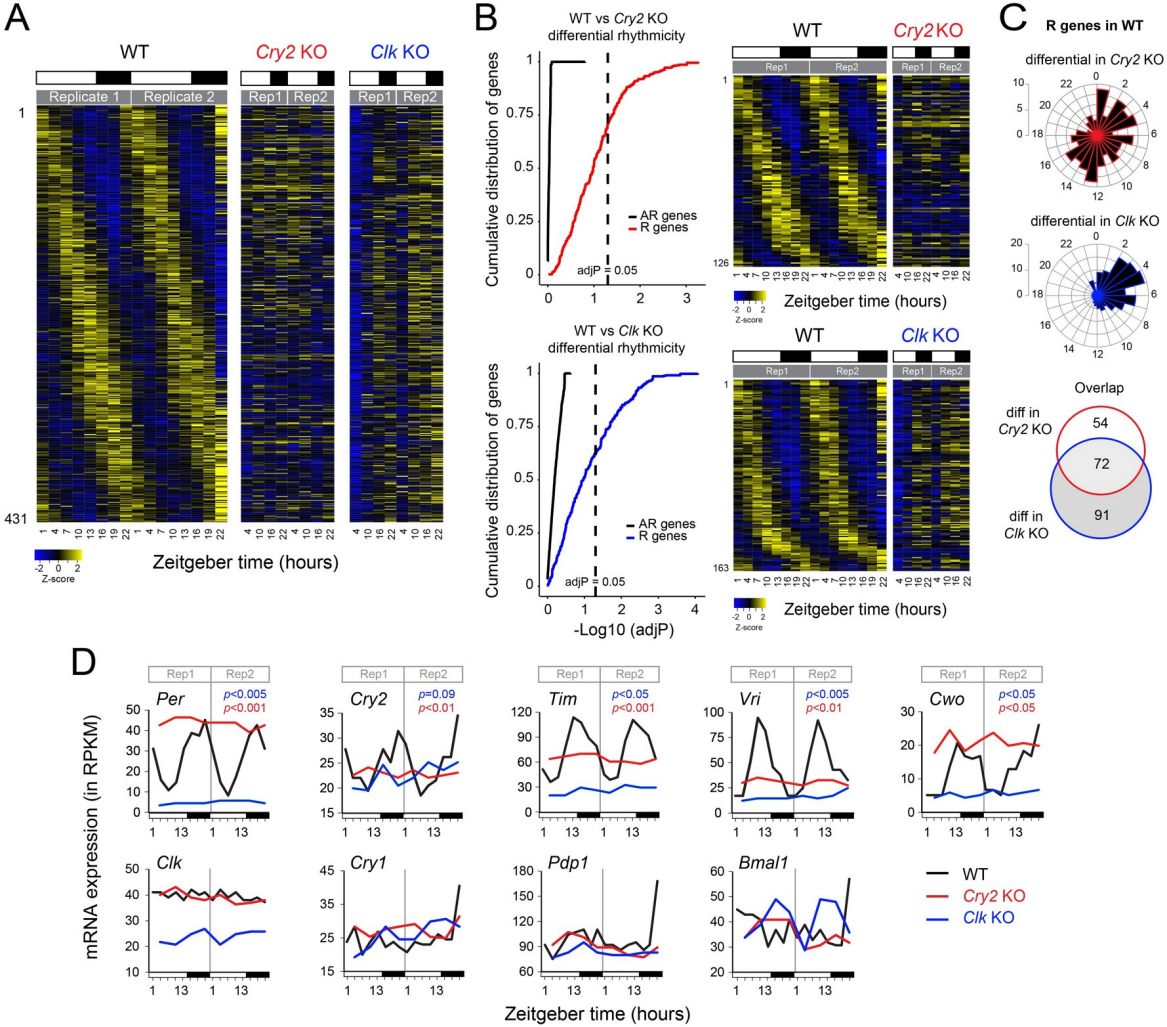
Twenty four-hour rhythms in mRNA abundance in the brain of monarch butterflies. A) *Top*, Heatmaps showing the relative RNA levels for genes rhythmically expressed in brains of wild-type, and their corresponding expression in brains of *Cry2* and *Clk* knockout monarchs, all entrained in 15:9 light:dark (LD) cycles. Z-scores of reads per base pair coverage are shown and plotted as heatmaps. Each column represents a single time point for a single replicate sampled over a 24-hour LD cycle starting at Zeitgeber Time (ZT) 1 in wild-type or ZT4 in *Cry2* and *Clk* knockouts. Two biological replicates are plotted consecutively. mRNAs are arranged by phase in wild-type and their order along the vertical axis is conserved for *Cry2* and *Clk* knockouts. White bars: light; black bars: dark. See S1 and S2 Tables for additional details. B) *Left*, Cumulative distributions of genes as a function of -Log10 of adjusted *p*-values (adjP) for differential rhythmicity in gene expression between wild-type and *Cry2* knockouts (*top*) and between wild-type and *Clk* knockouts (*bottom*). AdjP were obtained from p-values of the robust DODR [32] and corrected for multiple testing using the Benjamini-Hochberg method. Cumulative distributions for genes arrhythmic in wild-type (AR; black lines) are shown as a control. Dashed lines represent adjP = 0.05. *Right*, Heatmaps showing the relative RNA levels for genes rhythmically expressed in brains of wild-type found to be differentially expressed in brains of *Cry2* knockouts (*top*) and *Clk* knockouts (*bottom*). See S3 and S4 Tables for details. C) Phase plots showing the phase distribution of rhythmic mRNAs differentially regulated in *Cry2* and *Clk* knockouts identified in B (*top* and *middle*, respectively) and their overlap (*bottom*). D) RNA-seq temporal gene expression profiles of core clock genes in brains of wild-type, *Cry2* knockouts and *Clk* knockouts. For each gene and each genotype, the two biological replicates are plotted consecutively. Black line: wild-type; red line: *Cry2* knockout; blue line: *Clk* knockout. mRNA expression levels are expressed in reads per kilobase of transcript per million reads mapped (RPKM). *Per*, *period*; *Cry2*, *cryptochrome 2*; *Tim*, *timeless*; *Vri*, *vrille; Cwo*, *clockworkorange*; *Clk*, *clock*; *Cry1*, *cryptochrome 1*; *Pdp1*, *par domain protein 1*; *Bmal1*, *brain and muscle Arntl-like 1*. White bars: light; black bars: dark. See S1 Fig for qRT-PCR validation.

To determine the genes expressed rhythmically in the brain that may regulate physiological and behavioral rhythms in the monarch butterfly, we profiled genome-wide gene expression in the brains of wild-type adults collected every 3 hours in a 15-hr light:9-hr dark (LD) cycle using RNA-sequencing, with two independent replicates per time point. LD conditions were chosen instead of constant dark conditions because they reflect natural environments that drive diurnal rhythms of monarch activity and the 15:9 LD regime was used because it is the ecologically relevant photoperiod experienced by summer reproductive monarchs, and the condition under which we maintain our colony in the laboratory. Expression levels for each gene were examined for rhythmic variation using RAIN [30] and MetaCycle [31], and genes with a maximum/minimum fold-change ≥ to 1.3 and an adjusted *p* value (*i*.*e*., corrected for multiple testing) ≤ 0.05, as defined by either method, were considered rhythmic (see Methods for details). Using these criteria, we identified 431 rhythmic genes with peaks of expression distributed throughout day and night in the monarch brain (Fig 1A; S1 and S2 Tables). To determine whether the rhythmic expression of these genes was dependent on core clock genes, we also profiled genome-wide temporal expression patterns every 6 hours in 15:9 LD cycle in the brains of two monarch knockout strains bearing a non-functional circadian clock, lacking either a functional circadian activator CLK or a functional circadian repressor CRY2 [4,5]. An analysis of differential rhythmicity between rhythmic gene expression in wild-type and gene expression in clock-deficient mutants using DODR [32] revealed that the expression of most rhythmic genes in wild-type monarchs was affected in *Cry2* and *Clk* knockouts, and that this effect was significantly higher than background (calculated using genes arrhythmically expressed in brains of wild-type) (Fig 1B). Consequently, only genes with an adjusted p-value cutoff of ≤ 0.05 were considered in subsequent analysis. Under these stringent conditions, the expression of 126 and 163 rhythmic genes was respectively affected in *Cry2* and *Clk* knockouts (Fig 1B; S3 and S4 Tables), 72 of which overlapped in both datasets (Fig 1C; S5 Table). Interestingly, while the expression of the rhythmic genes differentially regulated in *Cry2* knockouts peaked throughout the day, the genes differentially regulated in *Clk* knockouts mostly peaked during the first half of the light phase (Fig 1C).

Among the rhythmic genes identified, we found the CLK:BMAL1 direct target core clock genes *Per* and *Tim*, which exhibited similar profiles to those previously shown by qPCR in wild-type monarch brains [5], as well as *Cry2* (Fig 1D). Some clock genes such as *vrille* (*Vri*) and *clockwork orange* (*Cwo*), whose products respectively function in *Drosophila* as a regulator of *clk* expression [33,34] and as a repressor of CLK-mediated activation [35–38], were also rhythmically expressed (Fig 1D). The rhythmicity and phase of *Per*, *Tim*, *Cry2*, *Vri* and *Cwo* expression in the brain of wild-type monarchs was confirmed by qRT-PCR on independent samples (S1 Fig), validating our RNA-seq data. The mRNA levels of other known clock genes did not however show cyclic expression in the monarch brain. This includes *Clk* and *Bmal1*, the blue-light photoreceptor *Cry1*, and the homologue of par domain protein 1 (*Pdp1*) that is required for behavioral rhythmicity in *Drosophila* [39] (Fig 1D).

If the rhythmic expression of core clock genes was solely controlled by the rhythmic activation of CLK:BMAL1, these genes would be predicted to be expressed at constitutively low levels in the absence of transcriptional activation (*i*.*e*., in *Clk* knockouts) and at constitutively high levels in the absence of transcriptional repression (*i*.*e*., in *Cry2* knockouts). As expected, we found that all clock genes rhythmically expressed in wild-type brains were expressed at constitutive low levels in the brain of *Clk* knockouts (Fig 1D). However, in the brains of *Cry2* knockouts, only *Per* and *Cwo* were found expressed at constitutive high levels (Fig 1D); *Vri* was expressed at constitutively low levels and *Tim* at mid-levels (Fig 1D), suggesting a complex regulation of their expression, either through the action of other factors on the regulation of their transcription or via post-transcriptional events.

Similar to rhythmic transcriptome analysis in brains of two other insects, *Drosophila* and the mosquito *Anopheles gambiae* [21,40–42], we found that rhythmic genes in the monarch brain belong to diverse biological processes that include transmembrane transport, several metabolic processes, and regulation of DNA binding (S2 Fig; S6 Table). KEGG pathway enrichment analysis further revealed that besides circadian rhythms, glycolysis, the biosynthesis of amino acids, carbohydrate metabolism, and other metabolic pathways were among the most enriched rhythmic pathways in the monarch brain (S2 Fig; S7 Table).

### Genes involved in glucose metabolism show coordinated rhythmic expression in the monarch brain

**Fig 2:**
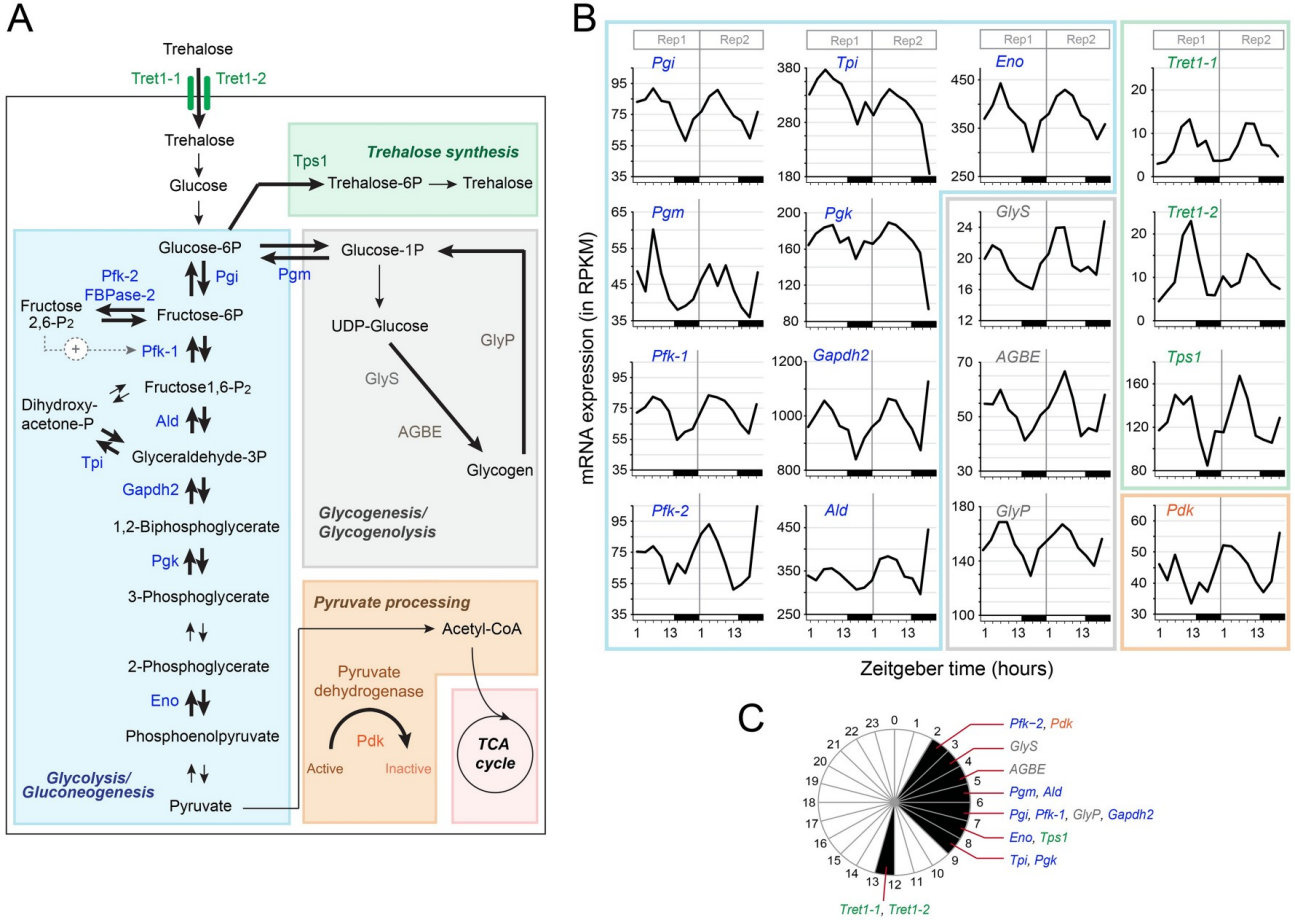
Coordinated rhythmic expression of key genes of the trehalose, glucose and glycogen metabolism pathways in the monarch brain. A) Key steps in trehalose transport and synthesis, glycolysis/gluconeogenesis, glycogenesis/glycogenolysis, and pyruvate processing pathways, and enzymes expressed rhythmically in the monarch brain (shown in color and bolded arrows). *Tret1*, *trehalose transporter* (*Tret1-1*, DPOGS215159; *Tret1-2*, DPOGS215160); *Tps1*, *trehalose-6-phosphate synthase* (DPOGS212595); *Pgi*, *phosphoglucose isomerase* (DPOGS210295); *Pfk-2/FBPase-2*, *6-phosphofructo-2-kinase/fructose 2*,*6 biphosphatase* (DPOGS215489); *Pfk-1*, *6-phosphofructokinase* (DPOGS203810); *Ald*, *aldolase* (DPOGS206959); *Tpi*, *triose phosphate isomerase* (DPOGS200089); *Gapdh2*, *glyceraldehyde 3 phosphate dehydrogenase 2* (DPOGS215460); *Pgk*, *phosphoglycerate kinase* (DPOGS213064); *Eno*, *enolase* (DPOGS207764); *Pdk*, *pyruvate dehydrogenase kinase* (DPOGS210186); *GlyS*, *glycogen synthase* (DPOGS214402); *AGBE*, *1*,*4-alpha-glucan branching enzyme* (DPOGS215494); *GlyP*, *glycogen phosphorylase* (DPOGS205027); *Pgm*, *phosphoglucose mutase* (DPOGS209508). B) Temporal expression profiles of the corresponding enzymes in the brain of wild-type monarchs in 15:9 LD. Two biological replicates are plotted consecutively. White bars: light; black bars: dark. See S1 Fig for qRT-PCR validation. C) Phase plot showing the expression of the genes in B in 1-hour bins.

Rhythmic expression of genes involved in metabolic pathways are thought to temporally orchestrate metabolic processes over the course of the 24-hour day [43,44]. In the monarch brain, we found that many genes encoding key enzymes and regulatory proteins involved in glucose metabolism, which is essential to fuel basic brain physiology, were expressed rhythmically (Fig 2A and 2B). Two trehalose transporters, Tret1-1 and Tret1-2, which act in glial cells to uptake trehalose (the main metabolite supplying energy in insects) and provide the brain with energy and protection from neurodegeneration [45], were rhythmically expressed with a peak of expression during the day (Fig 2B and 2C). To provide the brain with energy and nurture neurons, trehalose must be metabolized through glycolysis, which is toxic in neurons [46] but essential in glia [45]. Consistent with the idea that glial glycolysis is rhythmically regulated upon conversion of trehalose to glucose, several genes involved in glycolysis were also rhythmically expressed with peaks of expression within a 4-hr window in the middle of the day (Fig 2B and 2C). These included genes encoding (i) the enzyme involved in the rate-limiting step of glycolysis, 6-phosphofructokinase (*Pfk-1*), and (ii) other enzymes in the glycolytic pathway such as phosphoglucose isomerase (*Pgi*), aldolase (*Ald*), triose phosphate isomerase (*Tpi*), glyceraldehyde 3 phosphate dehydrogenase 2 (*Gapdh2*), phosphoglycerate kinase (*Pgk*), and enolase (*Eno*) (Fig 2A and 2B). Interestingly, genes encoding two regulatory proteins, 6-phosphofructo-2-kinase/Fructose 2,6 biphosphatase (*Pfk-2/FBPase-2*), which activates Pfk-1 to increase the glycolytic rate, and pyruvate dehydrogenase kinase (*Pdk*), which inhibits the activity of pyruvate dehydrogenase blocking the entry of pyruvate into the TCA cycle, were found to cycle in phase with one another (Fig 2B and 2C). These results strongly suggest that pyruvate catabolism is inhibited in phase with the regulation of glycolysis in glia.

Among genes expressed rhythmically with a peak of expression in the middle of the day, we also found three mRNAs of key players of glycogenesis/glycogenolysis, glycogen synthase (GlyS) and 1,4-alpha-glucan branching enzyme (AGBE), which generate highly branched glycogen molecules to store glucose for quick energy use, and glycogen phosphorylase, the rate-limiting step in glycogenolysis (Fig 2A, 2B and 2C). Interestingly, phosphoglucose mutase (Pgm), which links glycolysis to glycogenesis (Fig 2A), also cycled in a phase similar to those of genes regulating both pathways (Fig 2B and 2C). Finally, a gene involved in *de novo* synthesis of trehalose, *trehalose 6-phosphate synthase* (*Tps1*) was also rhythmically expressed in the monarch brain with a peak of expression during the day (Fig 2A, 2B and 2C), suggesting a possible local rhythmic synthesis of trehalose.

Importantly, the rhythmicity of the genes encoding key enzymes in glycolysis and glycogenesis appeared to be under the regulation of core clock components as their daily rhythms were, for the most part, disrupted in the brains of *Cry2* and *Clk* knockouts (S3 Fig). Together, our data provide evidence of a tight coordination in the temporal expression of genes involved in glucose metabolism in the insect brain, likely optimizing the timing at which energy substrates are produced to fuel the neurons and sustain their daily activities.

### Daily control of gene expression of neurotransmitters and GPCR signaling

**Fig 3:**
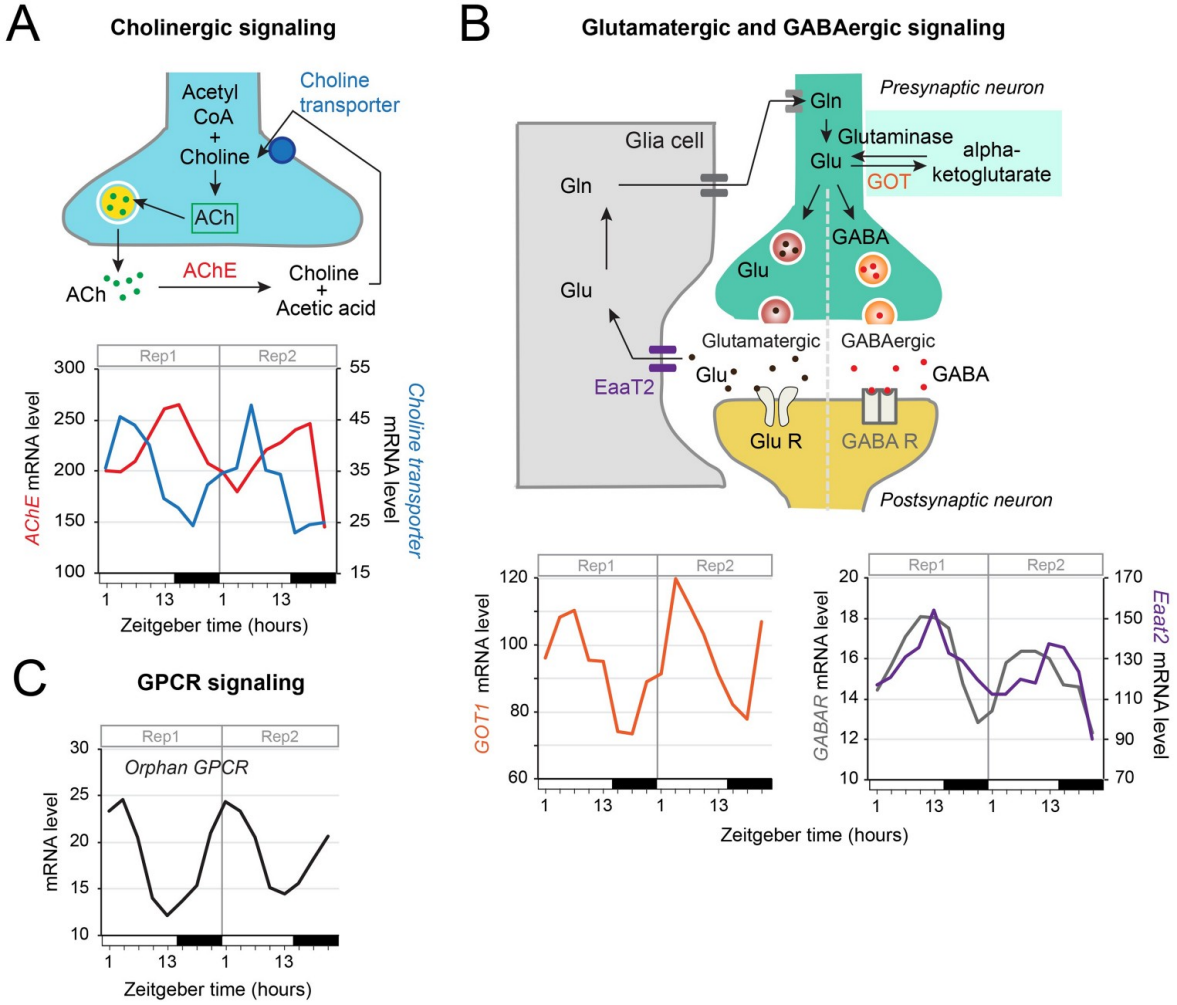
Diel rhythmic expression of genes involved in cholinergic, glutamatergic, and GABAergic neurotransmission, and GPCR signaling in the monarch brain. A) The neurotransmitter acetylcholine (ACh), synthesized from acetyl-CoA and choline, is released by presynaptic terminals to modulate the activity of target neurons [52]. In the synaptic cleft, acetylcholinesterase (AChE) breaks down ACh into acetate and choline, which is transported back into the presynaptic terminal through choline transporters. Antiphase rhythmic expression of *AChE* (DPOGS202609) and *choline transporter-like* (DPOGS213114) in 15:9 LD is shown. White bars: light; black bars: dark. B) Glutamate (Glu), the major excitatory neurotransmitter in the brain, is synthesized from glutamine (Gln) by glutaminase, and can be converted into alpha-ketoglutarate, an intermediary metabolite in the tricarboxylic acid cycle, by glutamate oxaloacetate transaminase (GOT). Glu is released by glutamatergic neurons to increase the neuronal excitability of post-synaptic neurons via glutamate receptors (GluR). Excess glutamate at the synapse is recycled by glial cells. Glu is taken up by these cells through an excitatory amino acid transporter (Eaat) and converted to Gln, which is then transported back into neurons [100]. In GABAergic neurons, the neurotransmitter GABA is synthesized from glutamate and released into the synaptic cleft where it inhibits the neuronal activity of post-synaptic neurons through its action on GABA receptors (GABAR). Rhythmic temporal expression of *GOT1* (DPOGS202178), *GABAR* (DPOGS204494) and *Eaat2* (DPOGS202815) are shown. C) Rhythmic control of a G-protein coupled receptor (GPCR) of unknown function (DPOGS205549) in the monarch brain. See S1 Fig for qRT-PCR validation.

Brain neuronal activity and communication within neural networks, which ultimately regulates physiology and behavior, occur via neurotransmission and G- protein coupled receptor (GPCR) signaling. In *Drosophila* and mammals, circadian rhythms are regulated by a number of neurotransmitters, neuropeptides and corresponding receptors that are under circadian clock control to temporally relay information across the clock network [47–51]. Not surprisingly, genes involved in cholinergic, glutamatergic, GABAergic and GPCR signaling were also found rhythmically expressed in the monarch brain (Fig 3). However, we noted interesting features of regulation of gene expression consistent with a temporal separation of chemically antagonistic processes.

mRNAs encoding acetylcholinesterase (AChE), the enzyme that breaks down acetylcholine (ACh) released by presynaptic terminals to decrease Ach signaling [52], and the choline transporter, which transports choline in the presynaptic terminal for the synthesis of ACh, were expressed rhythmically in anti-phase to one another (Fig 3A). Rhythms of *choline transporter* were abolished in the brains of *Cry2* and *Clk* knockouts (S4A Fig), and those of *AchE* were abolished in the brains of *Cry2* knockouts (S4A Fig). These data suggest a temporal partitioning of the synthesis and degradation of ACh over the course of the day that could be controlled by the circadian clock.

Genes involved in the recycling and reception of the neurotransmitters glutamate and γ-aminobutyric acid (GABA), which are known to drive circadian rhythms in *Drosophila* and mammals [53,54], were also found rhythmically expressed in the monarch brain. In neurons, glutamate levels can be decreased through its conversion to alpha-ketoglutarate by the enzyme glutamate oxaloacetate transaminase (GOT) (Fig 3B). We found that mRNA levels of GOT cycled in the monarch brain, with a peak of expression during the day (Fig 3B), and that the rhythms were disrupted in clock-deficient monarch strains (S4B Fig). Glutamate and GABA modulate the neuronal excitability of post-synaptic neurons through their respective action on glutamate receptors and GABA receptors (GABAR). Extracellular glutamate at the synapse is recycled by increased glial activity of glutamate transporters (excitatory amino acid transporters; EaaT) [55]. Interestingly, *GABAR* and *EaaT2* mRNA levels were also rhythmic in the monarch brain (Fig 3B) and significantly disrupted in *Cry2* and *Clk* knockouts, with the exception of *GABAR* in *Cry2* knockout where significance was not reached despite an apparent loss of rhythm (S4B Fig). Together, these data suggest that the circadian clock may rhythmically regulate neuronal excitability by modulating both the expression of genes involved in glutamate clearance at the synapse and of GABA receptors. Finally, we also found daily rhythms in mRNA levels of a G-coupled protein receptor (GPCR) of unknown function (Fig 3C) that are abolished in *Cry2* and *Clk* knockouts (S4C Fig), suggesting that it could be involved in modulating circadian behaviors.

### Chromatin accessibility measured by ATAC-seq is not clock-dependent in the monarch brain

Similar to *Drosophila* [27], rhythmic genes cycled in the monarch brain with phases of expression distributed throughout the day (Fig 1A). How the expression of these rhythmic genes is temporally orchestrated is however not fully understood. As shown in flies and mice, both transcriptional and post-transcriptional processes contribute to the generation of rhythmic mRNA levels [56–60]. Transcriptional regulatory mechanisms such as rhythmic accessibility of DNA regulatory elements [22] or rhythmic activities of distinct TFs [61–64] have been shown to control diurnal rhythms in transcription and could also contribute to the generation of different phases of gene expression in the monarch brain.

**Fig 4:**
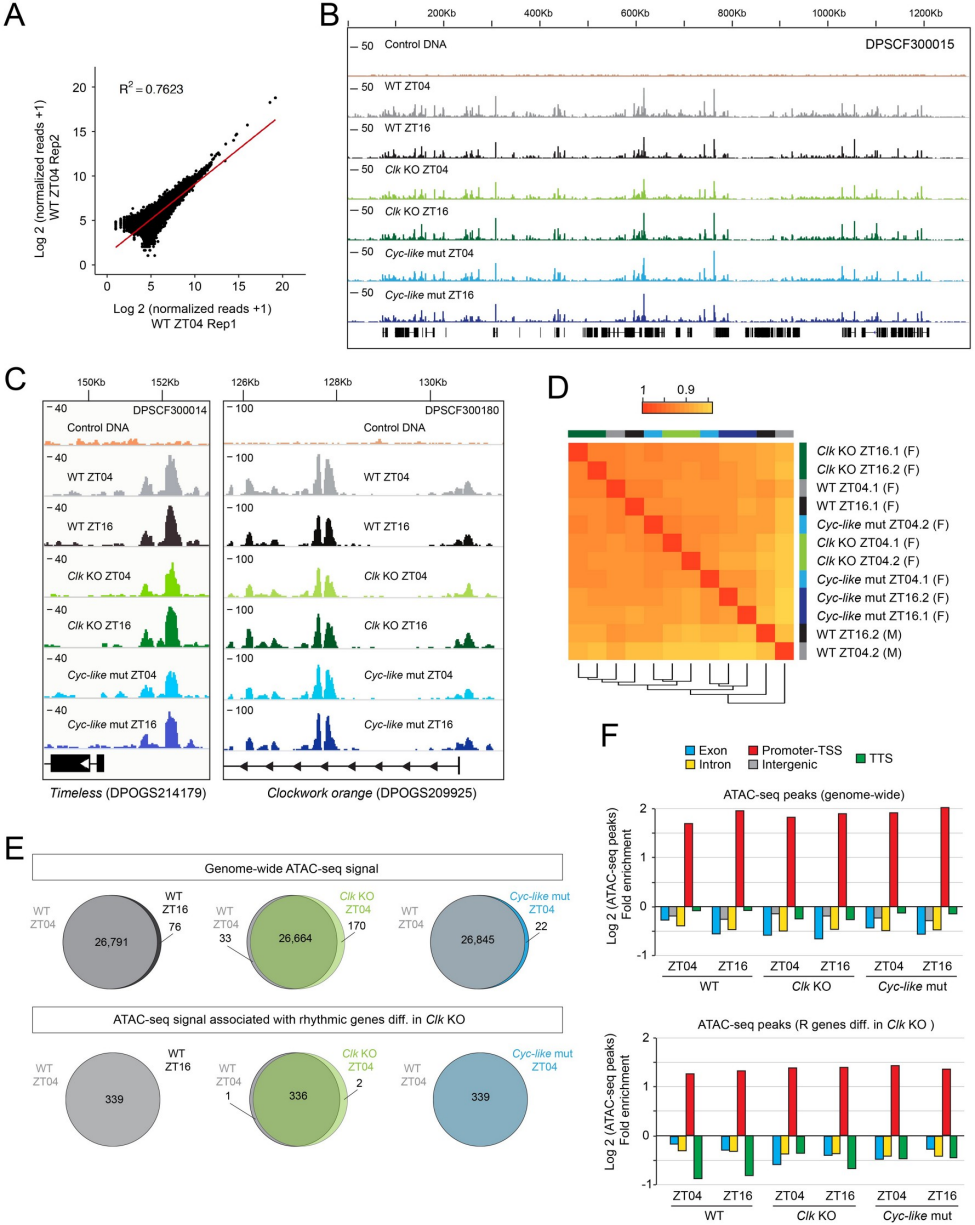
ATAC-seq captures chromatin accessibility in brains of wild-type monarchs and monarchs impaired for clock function. A) Correlation plot of two biological replicates showing normalized read counts in wild-type at ZT04. Correlation plots for all conditions tested are shown in S5A Fig. Correlations varied from 0.76 to 0.85. B) Visualization of ATAC-seq signal between genotypes and time points at scaffold DPSCF300015 showing no substantial gross change in chromatin accessibility in adult brain. ATAC-seq peaks from biological replicates are merged. WT, wild-type; *Clk* KO, *Clk* knockouts; *Cyc-like*, *Bmal1* mutants lacking the C-terminal transactivation domain [6]. C) Representative ATAC-seq tracks in genomic regions of clock genes (*timeless* and *clockwork orange*) showing the lack of differential ATAC-seq peaks between time points and genotypes. D) ATAC-seq signal within consensus ATAC-seq peaks was compared between all samples using Pearson’s correlation to cluster samples. Replicates are noted as numbers following each genotype and time point. F: female; M: male. E) Venn diagrams representing lack of differences in ATAC-seq signal within consensus genome-wide ATAC-seq peaks (*top*) and ATAC-seq peaks associated to rhythmic genes differentially regulated in *Clk* knockouts (*bottom*) between ZT04 and ZT16 in wild-type brains and between genotypes at ZT04 (fold-change cutoff > 1.3). The complete list of cross-comparisons is provided in S9 Table. F) Log2 fold enrichment of ATAC-seq peaks within different genomic regions in the monarch genome (*top*) and within rhythmic genes differentially regulated in *Clk* knockouts (*bottom*) for each genotype at ZT04 and at ZT16. Except for intergenic regions, genomic features are defined within -1Kb of the transcription start site (TSS) and +1Kb of the transcription termination site (TTS). Fold enrichment is calculated as the number of peaks per genomic regions/total number of peaks relative to the length of the genomic regions/total length.

To investigate whether rhythmic chromatin accessibility plays a role in the control of rhythmic gene expression in the monarch, we profiled open chromatin regions using ATAC-seq in the brains of wild-type monarchs and of two mutants deficient in circadian activation (*Clk* knockout and *Cyc-like*, a Bmal1 mutant lacking the C-terminal transactivation domain that mimics the *Drosophila cycle* gene [6]) at Zeitgeber Time 04 (ZT04; *i*.*e*., 4 hours after lights on) and at ZT16, with two independent replicates per time point (Fig 4). ZT04 was chosen as the day time point because it corresponds to the trough of *Per* and *Tim* RNA expression levels and thus the likely time at which transcription is activated for these genes, and ZT16 was chosen as the night time point such that the two would be separated by 12 hours. Across all samples, 13,555 to 23,222 ATAC-seq peaks were identified throughout the genome (S8 Table). These numbers are within the same order of magnitude as that obtained in *Drosophila*, which harbors a comparable genome size (~138 Mb for *Drosophila* versus ~249 Mb for monarch) [65]. We also found good replicate concordance of the ATAC-seq peaks between biological replicates (Fig 4A and S5A Fig; *R*^*2*^ ranging from 0.76 to 0.85). As expected, Tn5 tagmentation of purified naked genomic DNA from brain did not display characteristic ATAC-seq peaks (Fig 4B). These results strongly indicate that ATAC-seq can reliably and reproducibly measure chromatin accessibility in the monarch brain. Visual inspection of the ATAC-seq peaks from different genotypes and time-of-day revealed broadly similar profiles of chromatin accessibility (Fig 4B), even in regions associated to clock genes that display robust rhythms of expression (Fig 4C; see Fig 1D for mRNA expression patterns). A hierarchical clustering of consensus ATAC-seq peaks for all genotypes and time points also showed that they were strongly correlated with one another (Fig 4D). The lack of significant differences in ATAC-seq peaks was further validated by quantifying differential Tn5 integration signals using DESeq2 [66] with Log2 fold-change > 0.3785 and FDR < 0.05 as thresholds. Even at this relatively low fold-change cutoff, less than 0.64% of ATAC-seq peaks genome-wide or associated with rhythmic genes differentially regulated in *Clk* knockouts were found to be significantly altered between time-of-day (ZT04 vs. ZT16) and genotypes (wild-type vs. clock impaired mutants) (Fig 4E; S9 Table). While a lack of statistically significant differences is not evidence of absence of differences, previous reports showing that ATAC-seq is sensitive enough to identify differences in chromatin accessibility in *Drosophila* [67] support the idea that there is no significant temporal regulation of significant opening/closing of the chromatin in the monarch brain. To further characterize our ATAC-seq datasets, we mapped the genomic location of each ATAC-seq peak and found that genome-wide peaks were enriched by ~ 3 to 4-fold in promoter regions (Fig 4F). A similar enrichment was found for peaks in the promoter regions of genes rhythmically expressed in the brain and differentially regulated in *Clk* knockouts (Fig 4F). Many genome-wide peaks were also found in introns and intergenic regions, as well as in exons and transcription termination sites (S5B Fig), likely representing the full set of enhancer elements in the monarch brain.

### Clock-dependent temporal regulation of transcription factor occupancy in brains of wild-type monarchs

**Fig 5:**
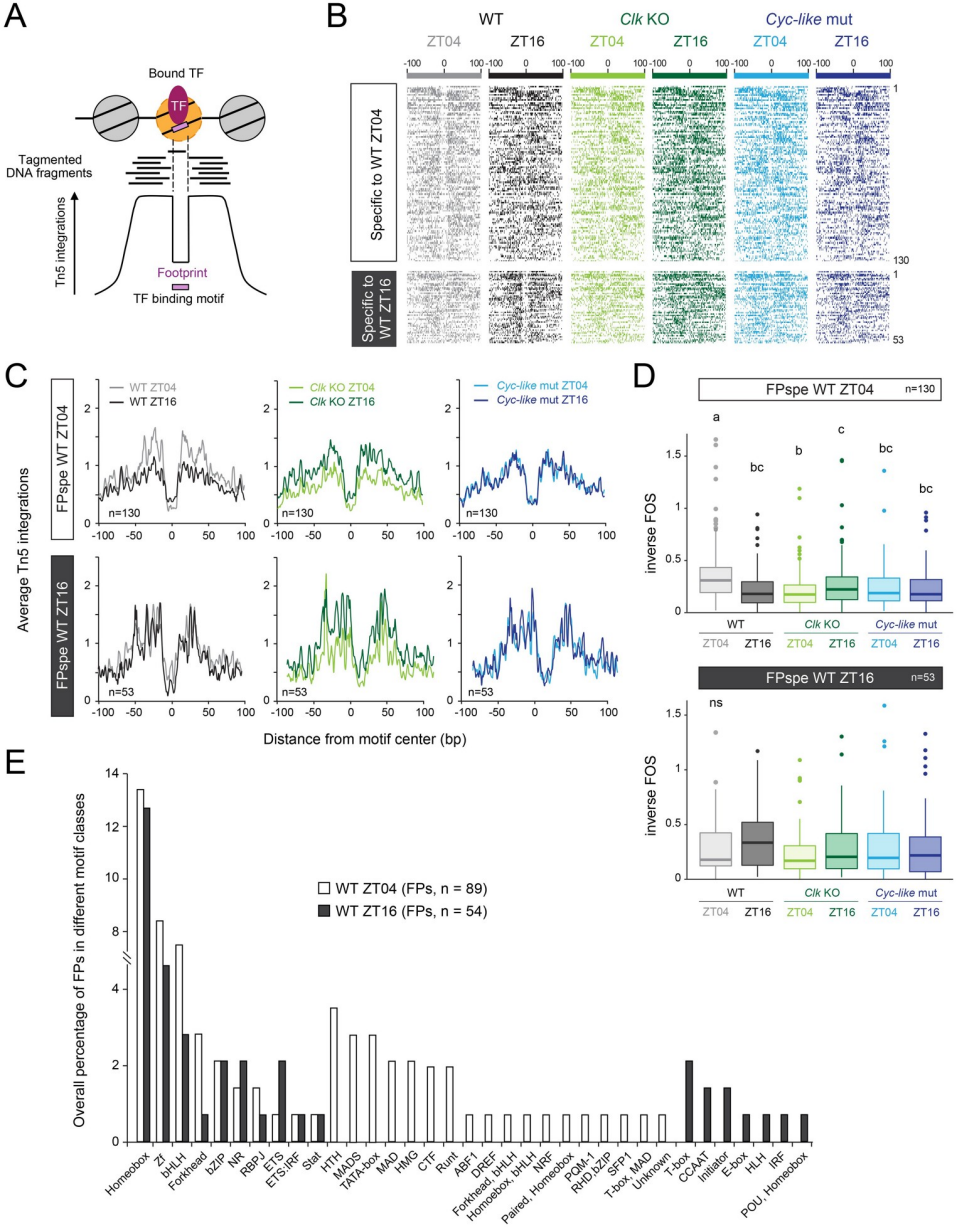
The depth of transcription factor (TF) footprints is temporally regulated in brains of wild-type monarchs and dependent on circadian activators. A) Within an accessible regulatory chromatin region, putative TF footprints may be detected as a narrow region that is locally protected from Tn5 integration. The identity of the TFs is inferred from binding motifs present in the DNA sequences of the footprints. B) ATAC-seq signals centered on the footprints detected within accessible chromatin of rhythmic genes differentially expressed in *Clk* knockouts in wild-type at ZT04 and at ZT16, as well as in the same regions in *Clk* knockouts and *Cyc-like* mutants. ATAC-seq peaks are grouped by the time of day at which the peaks are found to be specific in wild-type (ZT04 versus ZT16). C) Average profiles of Tn5 integrations centered on footprints specific to ZT04 or to ZT16 in wild-type, and in the same regions in *Clk* knockouts and *Cyc-like* mutants at both time points. Profiles were smoothed using a 3-bp rolling average. D) Box plots representing values of inverse footprint occupancy score (FOS) calculated for the footprints specific to wild-type at ZT04 (*top*) and specific to wild-type at ZT16 (*bottom*) in wild-type, *Clk* knockouts and *Cyc-like* mutants at ZT04 and at ZT16. Groups with different letters are statistically different. *P* < 0.05, Kruskal-Wallis test followed by Dunn test; ns: no statistically significant difference. E) Distribution of motif classes enriched in TF footprints specific to ZT04 (white) and ZT16 (gray) in wild-type. The number of each motif class was normalized to the number of all motif classes in TF footprints and expressed as a percentage. Motif classes are shown by order (from left to right) based on their occurrence in (1) both groups (white and gray bars), (2) ZT04 only, and (3) ZT16 only.

The lack of cyclic chromatin accessibility as measured by ATAC-seq does not exclude the possibility that the DNA regulatory elements within ATAC-seq peaks could be involved in driving rhythmic gene expression through the rhythmic binding of TFs. In addition to identifying chromatin accessibility, ATAC-seq can also be used to reveal DNA footprints, *i*.*e*. genomic regions protected from Tn5 integration because of DNA-bound proteins like TFs [68,69] (Fig 5A). To test whether rhythms in TF occupancy within DNA regulatory elements in promoters and enhancers could underlie rhythmic gene expression, we performed a footprinting analysis within the open chromatin of 339 ATAC-seq peaks associated with the 163 rhythmic genes differentially regulated in *Clk* knockouts using the Wellington TF footprinting algorithm [70]. We detected 202 and 125 statistically significant footprints in brains of wild-type monarchs at ZT04 and at ZT16, respectively (p-value ≤ 10^−10^; FDR ≤ 0.01). Of these, 130 and 53 were respectively specific to ZT04 and to ZT16 (Fig 5B). Most of these time point-specific footprints exhibited footprint signals below the statistical threshold at the other time point rather than being completely absent. This therefore suggested that the depth of TF footprints could be regulated in a time-of-day dependent manner in monarch brains. To test this idea, we averaged the signal of footprints specific to a given time point and compared it to the average signal observed at the other time point (Fig 5C and 5D). Averaged footprint signal and quantification confirmed a time-of-day dependent regulation of TF binding. For TF footprints specific to ZT04, we observed a significant increase in footprint depth at ZT04 relative to ZT16 (Fig 5D) that appeared to be caused by both increased Tn5 integration in regions directly flanking the footprint and decreased Tn5 integration within the footprint (Fig 5C). These results suggest that TF binding at ZT04 may occupy their DNA binding sites with a longer residence time and may also increase to some extent chromatin accessibility in their immediate surrounding regions. In contrast, TF footprints specific to ZT16 did not exhibit a significant increase in footprint depth at ZT16 compared to ZT04 (Fig 5C and 5D). To verify that time-of-day dependent TF binding is clock-dependent, we measured the footprint signal at the same genomic regions in brains of *Clk* knockouts and *Cyc-like* mutants at ZT04 and ZT16 (Fig 5B, 5C and 5D). We found that the time-of-day differences in TF footprint signal specific to ZT04 observed in wild-type monarch brains were abolished in *Cyc-like* mutants, in which the footprint signal was not significantly different than the signal observed at ZT16 in wild-type (Fig 5C and 5D). Although the signal in *Clk* knockouts was not significantly different to the one observed at ZT16 in wild-type, there was a significant difference between time points, with a moderate increase at ZT16 compared to ZT04 (Fig 5C and 5D). Together, these data suggest that the time-of-day differences in TF binding found in wild-type monarch may underlie rhythmic gene expression. Furthermore, the differences in footprint signal between ZT04 and ZT16 suggested that distinct classes of TFs could differentially bind DNA at different times of the day. To identify these TF classes, we performed a motif enrichment analysis in footprints found specifically at ZT04 and at ZT16 in brains of wild-type monarchs. The analysis revealed a large number of predicted TF families preferentially binding at both and either time points (Fig 5E). Among the predicted TF classes, the bHLH, forkhead, HTH and MADS TF families appeared as among the most overrepresented in footprints at ZT04 in comparison to those at ZT16. These data suggest that TFs belonging to these families could be responsible for the increased DNA accessibility observed in footprints at ZT04.

## Discussion

Numerous studies over the past 15 years have profiled daily and circadian transcriptional rhythms in various organisms and tissues [19–29]. Most genome-wide rhythmic gene expression studies performed to identify cycling mRNAs in the insect brain have however largely relied either on the use of whole heads [19,21,25–29], from which mRNAs derive mainly from the compound eyes, or on the use of specific subsets of clock neurons in the brain [40,71]. To date, a single study has profiled the circadian and daily transcriptome in the *Drosophila* brain [72]. With 217 robust rhythmic mRNAs whose expression was affected in *Cry2* and *Clk* knockouts, our daily transcriptome in the monarch brain provides a useful complement to that of *Drosophila* for future comparative approaches.

Consistent with previous reports [5,11,14], *Per* and *Tim* were identified in our RNA-seq study as core clock genes that cycle robustly in the monarch brain, validating the quality of the RNA-seq datasets. Our study extended the set of core clock genes with robust rhythms to *Vri*, *Cwo*, and to the mammalian-like circadian repressor *Cry2*, although the latter cycled with lower amplitude. While all cycling core clock genes were expressed at constitutive low levels in *Clk* knockouts, as anticipated in the absence of activation, their expression in *Cry2* knockouts ranged from low to high levels, suggesting a complex regulation of their expression. Although the mechanistic underpinnings are not yet understood, this phenomenon is not without precedent as the levels of CLK-CYC direct targets in *tim* and *per Drosophila* mutants are also intermediate [25]. In contrast to the above-mentioned core clock genes, neither the circadian activators *Clk* and *Bmal1*, the blue-light photoreceptor *Cry1*, or *Pdp1* were expressed rhythmically in the monarch brain. Because the brain is a heterogeneous tissue, it is possible that these genes could be expressed both in non-clock cells and in a subset of clock cells in which they could oscillate. Alternatively, post-transcriptional and/or post-translational mechanisms may be responsible for their rhythmic function. Future studies using *in situ* hybridization in the brain and/or a population of homogeneous clock cells, such as the DpN1 monarch cell line containing a light-driven clock [14], could help distinguish between these possibilities.

A number of genes relevant to brain physiology were also found to be rhythmic in brains of wild-type monarchs. Similar to a study in the Antarctic krill [73], our data revealed a temporal regulation of key enzyme-encoding genes involved in glucose, trehalose and glycogen metabolism; however, the expression of these genes appeared to be coordinated in the monarch, peaking within a 7-hr window in the middle of the day. Although our data do not provide information on the rhythmic production of glycolytic metabolites, they indicate that fuel production in the brain is under tight rhythmic regulation at the level of gene expression, and likely increased during the monarch active phase. Pronounced rhythms in the abundance of metabolites and metabolic fluxes have however been recently proposed to emerge from phase lags rather than from coordinated expression of key enzymes and/or of regulatory proteins at the transcriptional levels [74]. This does not seem to be the case for glucose metabolism in the monarch brain because the genes that respectively encodes the main regulatory proteins Pfk-2 (which stimulates glycolysis) and Pdk (which blocks the entry of pyruvate into the TCA cycle) cycle in phase with one another. One interesting possibility could be that instead of being converted to acetyl-CoA, pyruvate is converted into lactate and alanine in brain glial cells, which once secreted, are taken up by neurons to fuel the neuronal TCA cycle and to generate the ATP needed for synaptic transmission, as previously shown in *Drosophila* [45]. If this is the case, the coordination of rhythmic gene expression in glial glucose metabolism could be a mechanism to fuel the neurons during the active phase of the monarch, *i*.*e*. during the day. Likewise, glycolysis and glycogenesis appeared to be temporally coordinated through phosphoglucose mutase. Because this enzyme acts in glycogenesis when glucose levels are high and in glycogenolysis when glucose levels are low, the temporal coordination of these pathways may serve as a homeostatic mechanism ensuring proper, rhythmic fuel production even in period of fasting. Importantly, we found that the rhythms of almost all of the genes involved in glucose metabolism were abolished in *Cry2* and *Clk* monarch mutants with impaired clock function. Together with a report showing circadian regulation of metabolic genes in *Drosophila* heads [75], our data indicate that the circadian clock likely plays an important role for regulating daily rhythm of glucose metabolism and glucose homeostasis in the insect brain.

By revealing anti-phase rhythms of expression of key genes involved in the transport/synthesis and degradation of Ach, which are dependent on the presence of both circadian activator and repressor, our data support the idea that the circadian clock also plays a role in temporally separating chemically antagonistic processes. Like ACh, GABA is a widespread neurotransmitter in the insect brain, and similar to *Drosophila* [71], a GABA receptor is expressed rhythmically in the monarch brain. Together, our findings that both cholinergic and GABAergic signaling are rhythmic and dependent on a functional circadian clock may suggest the existence of a more complex neuronal clock network than the four clock cells described in each hemisphere of the pars lateralis [14,76]. Developing CRISPR/Cas9-assisted methods to tag neurons *in vivo* would facilitate a comprehensive mapping of the neuronal clock network in the monarch brain.

Our study also establishes ATAC-seq as an applicable method for the discovery of both genome-wide accessible chromatin regions and TF binding in a non-traditional model organism for which species-specific antibodies against TFs are not available. Although thousands of genome-wide regulatory elements were identified in the monarch brain with ATAC-seq, a surprising result was the absence of a significant temporal change in chromatin accessibility between day and night, as well as between wild-type and clock impaired mutants. This was surprising because diurnal changes in chromatin accessibility measured by DNase-seq have recently been reported in the mouse liver [22]. The different nature of tissues sampled could account for this difference. If so, our data would support the idea that the chromatin accessibility landscape in the adult brain could be fully programmed and not sensitive to changes in light:dark conditions. Alternatively, the contrasting results between our study and the study in the mouse liver could stem from differences in sensitivity between ATAC-seq and DNase-seq approaches. Although DNase-seq could be a more sensitive approach to quantify diurnal changes in chromatin accessibility, applying it to samples like the insect brain may not be achievable due to the large amount of material required. Regardless of the reasons underlying the lack of diurnal changes in chromatin accessibility in our study, performing a TF footprinting analysis in accessible chromatin regions associated with genes rhythmically expressed revealed time-of-day regulation of TF occupancy in the brain of wild-type monarchs. Interestingly, time-of-day dependent TF occupancy was completely abolished in *Cyc-like* mutants that lack the transactivation domain necessary for CLK:BMAL1-mediated transcriptional activation [6]. The situation found in *Clk* mutants was slightly different in that time-of-day dependent TF occupancy was impaired, but the impairment consisted of a reversed trend compared to TF occupancy in wild-type. Because the CLK partner BMAL1 is present in this mutant, it is possible that in absence of a functional CLK, BMAL1 heterodimerizes with another bHLH-PAS domain-containing protein to activate transcription in a different phase than that of CLK:BMAL1. The juvenile hormone (JH)-receptor methoprene-tolerant (MET) could be a candidate, as it has been shown to form a heterodimer with BMAL1 in mosquitoes to activate the circadian transcription of JH-induced genes [77]. In addition, a motif enrichment analysis within open chromatin regions associated with rhythmic genes differentially expressed in *Clk* mutants revealed a large number of predicted TF families preferentially binding at either time point, indicative of the complexity of circadian regulation of gene expression in the monarch brain. Among the predicted TF classes, the bHLH, forkhead, HTH and MADS TF families appeared among the most overrepresented in footprints at ZT04 in comparison to those at ZT16. These data suggest that TFs belonging to these families could be responsible for the increased DNA accessibility observed in footprints at ZT04 in brains of wild-type monarchs, consistent with the reported pioneer activities of bHLH and forkhead TFs [78,79]. Together, these data show that ATAC-seq can be used in non-traditional organisms to not only identify open chromatin regions but also predict dynamic TF binding. It is of particular importance because the overall lack of species-specific antibodies against TFs in non-model organisms continues to preclude the systematic use of chromatin-immunoprecipitation to comprehensively identify TF binding sites.

Taken all together, our results represent the first analysis of daily transcriptome, DNA regulatory elements, and time-of-day dependent TF occupancy in the monarch butterfly brain. Given the central role of circadian clocks in the seasonal migration of this iconic insect, our datasets will be valuable resources to further our understanding of the molecular basis of seasonality and of migratory behavior.

## Methods

### Maintenance of monarch butterflies

Wild-type, *Cry2* knockout, *Clk* knockout and *Cyc-like* mutant monarch butterflies were raised in the laboratory on semi-artificial diet under 15-hour light, 9-hour dark (15:9 LD) conditions in Percival incubators at 25°C and 70% humidity, as previously described [4–6]. 15:9 LD was used because it is the ecologically relevant photoperiod experienced by summer reproductive monarchs, and the condition under which we maintain our colony in the laboratory. Adults were housed in glassine envelopes in the same lighting and temperature conditions, and were manually fed a 25% honey solution daily.

### RNA-sequencing experiments

Adult monarchs were entrained for a minimum of 7 days after eclosion in 15:9 LD cycles at 25°C and brains free of eye photoreceptors were dissected under a microscope in 0.5X RNA later (Ambion) to prevent RNA degradation, and stored at -80°C until use. For wild-type monarchs, three pooled brains were collected in two replicates at ZT1, ZT4, ZT7, ZT10, ZT13, ZT16, ZT19, and ZT22. Three pooled brains of *Cry2* and *Clk* knockouts were each collected in two replicates at ZT4, ZT10, ZT16, and ZT22. For each sample, total RNA was extracted using RNeasy Mini kit (Qiagen), polyA+ RNA was isolated from 2 μg of total RNA with NEBNext Oligo d(T) magnetic beads (New England Biolabs), and libraries were prepared using the NEBNext Ultra Directional RNA Library Prep kit for Illumina and NEBNext Multiplex Oligos (New England Biolabs) and amplified with 12 PCR cycles, following the manufacturer’s recommendations. Library quality and size distribution was verified on a Bioanalyzer, libraries were quantified by real-time quantitative PCR, and 16 libraries were mixed in equimolar ratios for multiplexing and sequenced on a Hi-seq 2500 (Illumina) using 50bp single end reads.

### RNA-sequencing data processing

The resulting sequencing files were checked for quality control and demultiplexed by the Texas A&M AgriLife Genomics and Bioinformatics Facility. Reads were mapped to the monarch genome (assembly v3; [80]) using TopHat2 [81] with parameters “—read-realign-edit-dist 2 -g 1 —b2-sensitive”. On average, ~88% of the reads were mapped uniquely to the genome even in absence of rRNA depletion (S10 Table). The total number of reads, mapped reads and mapping rate for each library are summarized in S10 Table. After mapping, gene expression levels were quantified in the brains of wild-type monarchs, *Cry2* knockouts and *Clk* knockouts at each time point and for each replicate using Cufflinks [82,83]. Only genes with three or more reads per kilo base per million mapped reads (RPKM) in at least one time point were classified as expressed and further considered for subsequent analysis.

### Identification of cycling mRNAs

To identify cycling mRNAs in the brain of wild-type monarchs, RAIN [30] and MetaCycle [31] were used with parameters “period = 24, deltat = 3, nr.series = 2” for RAIN and “adjustPhase =“predictedPer”, combinePvalue =“fisher”, timepoints = seq(1, 46, by = 3), minper = 24, maxper = 24” for MetaCycle. To consider oscillations determined by either method, the resulting p-values from RAIN and MetaCycle were combined using the minP method [84] and adjusted for multiple testing using the Benjamini-Hochberg (BH) procedure to control for false discovery rate (FDR). Genes were considered rhythmically expressed when meeting the following criteria: (1) adjusted p-value ≤ 0.05, and (2) fold-change (maximal/minimal RPKM expression values within a time series) ≥ 1.3. Of the 15,130 genes in the monarch genome, ~ 69% (10,412) were expressed in the brain of wild-type monarchs, and ~4% (431) of these were determined as being rhythmically expressed. To determine if the oscillations of the rhythmic genes identified in wild-type were altered in *Cry2* and *Clk* knockouts, a differential rhythmicity analysis was performed using robust DODR method [32]. After adjusting the p-values using the BH procedure, 126 and 163 genes rhythmically expressed in wild-type were respectively found to be differentially expressed in *Cry2* and *Clk* knockouts. Phases were estimated using the R package for harmonic regression [85]. Heatmaps and phase plots were respectively generated using the R packages gplots [86] and ggplot2 [87]. Gene ontology (GO) terms for biological processes and KEGG pathway enrichment analysis were performed using Metascape (http://metascape.org; [88]) against the expressed genes in the wild-type monarch brains as background. Sequencing data are available at https://www.ncbi.nlm.nih.gov/geo/query/acc.cgi?acc=GSE122447 (Accession number GSE122442).

### Real-time qPCR

Brains from adult wild-type monarch butterflies entrained to seven 15:9 LD cycles after eclosion were dissected every 3-hours over a 24-hour day starting at ZT1. Dissections were performed in 0.5X RNA later (Ambion) to prevent RNA degradation, and brains free of eye photoreceptors were immediately frozen and stored at -80°C until use. Total RNA was extracted using an RNeasy Mini kit (Qiagen), treated with RQ1 DNase (Promega), and random hexamers (Promega) were used to prime reverse transcription with Superscript II Reverse Transcriptase (Thermo Scientific), all according to the manufacturers’ instructions. Quantifications of gene expression were performed on a QuantStudio 6 Flex Real-Time PCR System (Thermo Scientific) using iTaq Universal SYBR Green Supermix (Bio-Rad), as previously described [4]. Monarch *Per*, *Tim*, and *Rp49* expression levels were quantified using previously validated primers [4]. Monarch *Cry2*, *Vri*, *Cwo*, *GOT1*, *GPCR*, *AGBE*, *Tps1*, and *Eno* expression levels were quantified using the following primers: *Cry2F*, 5’-TGGCTCTCATGCTCGTCTTTC-3’; *Cry2R*, 5’-ACCGCACTGGACAGTAGCAAT-3’; *VriF*, 5’-CGGACAGCGTAAGCAGAGAGA-3’; *VriR*, 5’-TCCCAGTAACCGTCGTCCTT-3’; *CwoF*, 5’-GCGCGCGCGCTTCAC-3’; *CwoR*, 5’-TCGATGCAGGGTTGGAAGTT-3’; *GOT1F*, 5’-CACAACCCCACAGGCATAGA-3’; *GOT1R*, 5’-CCATGACATCAGCGATCTTCTC-3’; *GPCRF*, 5’-GGGTACGAGCGGTATAGACATTG-3’; *GPCRR*, 5’- CTGCAAAGGACACTGGTCGAT-3’; *AGBEF*, 5’-CGGATGGCTCGCATCAA-3’; *AGBER*, 5’-TTTGTCGCCTTCATGCTTACA-3’; *Tps1F*, 5’-GACGGCGGGAAAAACAGA-3’; *Tps1R*, 5’-GCCTTGAGGAACGCCTTCA-3’; *EnoF*, 5’-GACTGTCGACCGGCCAGAT-3’; *EnoR*, 5’-ATTTGGCGAGACGCTCAGA-3’. The near 100% efficiency of each primer set was validated by determining the slope of Ct versus dilution plot on a 3 x 10^4^ dilution series. Individual reactions were used to quantify each RNA level in a given cDNA sample, and the average Ct from duplicated reactions within the same run was used for quantification. The data for each gene at a given time-point were normalized to *Rp49* as an internal control, and normalized to the mean of one sample within a set for statistics. P-values were calculated using one-way ANOVA in R.

### ATAC-sequencing experiments

Adult monarchs were entrained for a minimum of seven days after eclosion in 15:9 LD cycles at 25°C and brains free of eye photoreceptors were dissected in ice-cold ringer’s solution. Three pooled brains of wild-type, *Clk* knockout, and *Cyc-like* mutant monarchs were each collected in two replicates at ZT04 and at ZT16. Each sample was resuspended twice in 600 μl of NP-40 lysis buffer (10mM Tris-HCl at pH 7.5, 10 mM NaCl, 3 mM MgCl_2_, and 0.1% NP-40). Crude nuclei were prepared by gently homogenizing the brains in an ice-cold 2-ml Dounce homogenizer with two strokes of a loose-fitting pestle. After centrifugation, the pellet was directly subjected to transposition by Tn5 transposase for 30 min at 37°C using the Nextera DNA Library Preparation kit (Illumina), and the tagmented DNA was then purified using a Zymo DNA Clean & Concentrator-5 kit, all according to a previously published protocol [69]. To generate a control naked DNA library, 1 ng of genomic DNA extracted with phenol/chloroform from six pooled wild-type brains was also subjected to transposition by Tn5 transposase and the tagmented DNA was purified following the same procedures. Barcoded libraries were PCR amplified, each using a common custom primer and a unique custom Nextera barcoded primer, as in [69]. For each library, after an initial round of five PCR cycles, the optimal number of PCR cycles needed to stop amplification prior to saturation was estimated by real-time quantitative PCR. Five to ten additional PCR cycles were then performed bringing the total number to 10 (for control DNA) and to 15 cycles (for other samples), and the libraries were purified using a Qiagen MinElute PCR purification kit. Library quality and size distribution was assessed on a Bioanalyzer, libraries were quantified by real-time quantitative PCR and mixed in equimolar ratios before sequencing on a single lane of Hi-seq 2500 (Illumina) using 50bp single end reads.

### ATAC-sequencing data processing: Identification, quantification, annotation and differential analysis of ATAC-seq peaks

The resulting sequencing files were checked for quality control and demultiplexed by the Texas A&M AgriLife Genomics and Bioinformatics Facility. After removing the adapter sequence using fastx clipper (hannonlab.cshl.edu/fastx_toolkit/) with options “-Q33 -n -v”, the clipped reads were mapped to the monarch genome (v3; [80]) using Bowtie2 [89] with parameters “—phred33—local”. Unmapped reads and mapped reads with mapping quality below 10 after sorting with SAMtools version 0.1.19 [90] were discarded. Mapped reads were adjusted such that those aligned to the positive strand and negative strand were shifted by +4 bp and –5 bp, respectively, as described in [69].

ATAC-seq peaks were called using MACS2 [91] with parameters “-q 0.01—nomodel—shift -100—extsize 200—keep-dup all”, using reads from naked DNA as control. For each genotype and time point, consensus peaks between biological replicates were generated by merging peaks with overlapping coordinates, using HOMER [92]. Pairwise comparison of the biological replicates was performed by quantifying the density of reads contained within consensus peaks in each replicate, using HOMER [92]. To compare replicates from all genotypes and time points to one another, peaks from all libraries were merged into a set of 37,642 consensus peaks. Read densities were quantified in each sample, and correlation was determined using the Pearson correlation coefficient [93]. Given that high level of reproducibility was found between biological replicates, ATAC-seq peaks from replicate libraries of wild-type, *Clk* knockouts and *Cyc-like* mutants at ZT04 and at ZT16 were merged by calling peaks on combined replicates using MACS2 [91] with the same parameters as described above. The peaks called, which ranged from 13,555 to 23,222, were used in subsequent analysis. With the exception of a few peaks that could not be annotated because they were located in scaffolds containing no annotated genes, all peaks in the genome were assigned using HOMER [92] based on their locations relative to a gene as follows: (1) promoter-TSS if present within –1kb to +100 bp of the transcription start site (TSS), (2) TTS if within -100 bp to +1kb of the transcription termination site (TTS), (3) exon if within any exon, (4) intron if within any intron, or (5) intergenic. Peaks associated with genes rhythmically expressed in wild-type and differentially expressed in *Clk* knockouts were determined from these annotations. Differential peak analysis between conditions was performed on sets of overall merged genome-wide peaks and peaks associated with genes rhythmically expressed in wild-type and differentially expressed in *Clk* knockouts using HOMER [92] and its R package DESeq2 [66] wrapper with cutoff thresholds for FDR of < 0.05 and log2 fold-change of > 0.3785 and < -0.3785. To visualize ATAC-seq peaks, bigWig files normalized to 10 million reads were generated from BAM files and visualized using the Integrative Genomics Viewer (IGV; [94,95]). Sequencing data are available at https://www.ncbi.nlm.nih.gov/geo/query/acc.cgi?acc=GSE122447 (Accession number GSE122445).

### Transcription factor footprinting of ATAC-seq peaks

Occupied TF DNA binding sites within ATAC-seq peaks associated with genes rhythmically expressed in wild-type and differentially expressed in *Clk* knockouts were identified in the brains of wild-type monarchs at ZT04 and at ZT16 using the Wellington TF footprinting algorithm in pyDNase [70] with p-value < 10^−10^ and FDR < 0.01. This program uses the imbalance in the reads aligned to the positive and negative strands surrounding the protein-DNA interactions to accurately predict which binding sites are protected from Tn5 integrations. Footprints specific to either time point were identified using BEDTools [96]. Footprints from each group were further analyzed in wild-type, *Clk* knockouts and *Cyc-like* mutants at ZT04 and at ZT16 using the dnase_to_treeview.py script in pyDNase [70] to obtain Tn5 integration counts from the center of the footprints flanked by 100 bp on either side. Visualization was performed by generating heatmaps using Java Treeview [97]. To determine the effect of clock disruption on time-of-day dependent footprints, average profiles were generated from the average of Tn5 integration counts for each bp of the 200 bp regions. Data smoothing was applied using 3-bp rolling averages.

To compare the depth of footprints found to be specific to ZT04 or specific to ZT16 in wild-type between wild-type, *Clk* knockouts and *Cyc-like* mutants at ZT04 and at ZT16, a footprint occupancy score (FOS) was quantified using the formula (*C* + 1)/*L* + (*C* + 1)/*R* [98]. *C* corresponds to the average of Tn5 integrations within the central region of the footprints where a transcription factor is directly engaged, while *L* and *R* correspond to the average of Tn5 integrations respectively on the left and right flanking regions on each side of the footprints. Since Wellington footprinting algorithm identified footprints with 11–25 nucleotides in length and flanked by 35 nucleotides on each side [70], the average of Tn5 cuts on both positive and negative strands corresponding to these lengths were used in the calculations. Data were represented as inverse FOS such that higher inverse FOS values indicate stronger footprints, *i*.*e*. a higher difference between the central and flanking regions of footprints. Inverse FOS values were visualized through box plots using ggplot2 [87] and the statistical analysis was performed using the base R function kruskal.test and the R package dunn.test [99].

### Enrichment of motif classes in TF footprints within rhythmic genes differentially expressed in *Clk* knockouts

Motif enrichment analysis was performed on TF footprints (FPs) located within peaks of genes rhythmically expressed in wild-type but differentially expressed in *Clk* knockouts that were specific to either ZT04 or ZT16 in wild-type monarch brains using findMotifsGenome script in HOMER [92] with parameters “-size given -mask”. For each motif, a fold-change > 1.5 was used as a measure of enrichment. Fold-change was expressed as the proportion of a given motif in FPs associated with rhythmically expressed genes over background, divided by its corresponding proportion in genome-wide FPs over background. Genome-wide FPs were identified using the combined 22,004 FPs at ZT04 and 14,101 FPs at ZT16. Background sequences were randomly selected from a pool of sequences that (1) did not contain sequences in which FPs were found, (2) did not contain repeats sequences, and (3) whose length and GC-content matched those of input FPs. Sequence-biases and plant-specific motifs were filtered out from the analysis. Enriched known motifs were assigned to the corresponding FPs using HOMER’s findMotifsGenome script [92] with option “-find <motif matrix file>”. In cases where several TFs belonging to the same class (*i*.*e*., TFs with similar DNA binding domains) were identified to occupy the same FP, only one was retained for further analysis. The percentage of motif classes and their distribution was calculated for FPs specific to ZT04 and to ZT16 in wild-type monarch brains.

## Supporting information

S1 Table Rhythmic genes identified by either RAIN or MetaCycle with adjusted *p*-value (adjP) ≤ 0.05 and fold-change (maximum/minimum expression values) ≥ 1.3. (DOCX)

S2 Table RPKM values of genes identified by either RAIN or MetaCycle to be rhythmic in wild-type monarchs, and the corresponding RPKM values in *Cry2* and *Clk* knockouts. (XLSX)

S3 Table Rhythmic genes in wild-type differentially expressed in *Cry2* knockouts with adjusted *p*-value (adjP) ≤ 0.05 from robust DODR method. (DOCX)

S4 Table Rhythmic genes in wild-type differentially expressed in *Clk* knockouts with adjusted *p*-value (adjP) ≤ 0.05 from robust DODR method. (DOCX)

S5 Table Rhythmic genes in wild-type that are differentially expressed both in *Cry2* knockouts and *Clk* knockouts with adjusted *p*-value (adjP1 for wild-type vs. *Cry2* knockouts, and adjP2 for wild-type vs. *Clk* knockouts) ≤ 0.05 from robust DODR method. (DOCX)

S6 Table Enriched Gene Ontology (GO) terms of biological processes for rhythmic genes in wild-type differentially expressed in (A) *Cry2* knockouts and (B) *Clk* knockouts. (DOCX)

S7 Table Enriched KEGG pathways for rhythmic genes in wild-type differentially expressed in (A) *Cry2* knockouts and (B) *Clk* knockouts. (DOCX)

S8 Table Number of ATAC-seq peaks identified for individual and merged replicates in wild-type, *Clk* knockouts (KO) and *Cyc-like* mutants (mut) at ZT04 and at ZT16. R: biological replicate.
(DOCX)

S9 Table Differential ATAC-seq peak analysis. Higher Tn5 integration depicts a Log2 fold-change > 0.3785 (fold-change > 1.3) and FDR < 0.05.
(DOCX)

S10 Table RNA-seq summary for wild-type, *Cry2* knockouts, and *Clk* knockouts. R: biological replicate.
(DOCX)

S1 Fig Validation of rhythmic gene expression of candidate genes by qRT-PCR. Diurnal expression of core clock genes (*top*) and a few other rhythmic candidate genes (*bottom*) in brains of wild-type monarchs entrained in 15:9 LD conditions. Values are mean ± SEM of six animals. Horizontal bars, day (white) and night (black). P-values, one-way ANOVA. *Per*, *period*; *Cry2*, *cryptochrome 2*; *Tim*, *timeless*; *Vri*, *vrille*; *Cwo*, *clockworkorange*; *AGBE*, *1*,*4-alpha-glucan branching enzyme*; *Tps1*, *trehalose-6-phosphate synthase*; *Eno*, *enolase*; *GOT1*, *glutamate oxaloacetate transaminase*; *GPCR*, *G-protein coupled receptor of unknown function*.
(TIF)

S2 Fig Enriched Gene Ontology terms (GO) of biological processes (A) and KEGG pathways (B) of rhythmic genes. GO and KEGG pathways for genes with rhythmic expression levels in wild-type but differentially expressed in *Cry2* and *Clk* knockouts, identified by Metascape at *p* <0.01, are shown in red and blue, respectively.
(TIF)

S3 Fig Temporal expression profiles of enzymes involved in key steps of the glycolysis, trehalose and glycogen metabolism pathways in brains of wild-type monarchs, *Cry2* knockouts and *Clk* knockouts entrained in 15:9 LD. For each gene and each genotype, two biological replicates are plotted consecutively. Black line: wild-type; red line: *Cry2* knockouts; blue line: *Clk* knockouts. mRNA expression levels are expressed in reads per kilobase of transcript per million reads mapped (RPKM). *Tret1*, *trehalose transporter* (*Tret1-1* and *Tret1-2*); *Tps1*, *trehalose-6-phosphate synthase*; *Pgi*, *phosphoglucose isomerase*; *Pfk-2/FBPase-2*, *6-phosphofructo-2-kinase/fructose 2*,*6 biphosphatase*; *Pfk-1*, *6-phosphofructokinase*; *Ald*, *aldolase*; *Tpi*, *triose phosphate isomerase*; *Gapdh2*, *glyceraldehyde 3 phosphate dehydrogenase 2*; *Pgk*, *phosphoglycerate kinase*; *Eno*, *enolase*; *Pdk*, *pyruvate dehydrogenase kinase*; *GlyS*, *glycogen synthase*; *AGBE*: *1*,*4-alpha-glucan branching enzyme*; *GlyP*, *glycogen phosphorylase; Pgm*, *phosphoglucose mutase*. *p*-values were obtained from *p*-values of the robust DODR [32] and corrected for multiple testing using the Benjamini-Hochberg method.
(TIF)

S4 Fig Temporal expression profiles of genes encoding proteins involved in cholinergic (A), glutamatergic and GABAergic (B) neurotransmission, and GPCR signaling (C) in brains of wild-type monarchs, *Cry2* knockouts and *Clk* knockouts entrained in 15:9 LD. For each gene and each genotype, two biological replicates are plotted consecutively. Black line: wild-type; red line: *Cry2* knockouts; blue line: *Clk* knockouts. mRNA expression levels are expressed in reads per kilobase of transcript per million reads mapped (RPKM). *p*-values were obtained from *p*-values of the robust DODR [32] and corrected for multiple testing using the Benjamini-Hochberg method.
(TIF)

S5 Fig Chromatin accessibility measured by ATAC-seq in brains of wild-type monarchs and monarchs impaired for clock function. A) Scatter plots showing the ATAC-seq signal correlation between biological replicates at ZT04 and at ZT16 in brains of wild-type, *Clk* knockout and *Cyc-like* mutant monarchs. B) Distribution of ATAC-seq peaks in the monarch genome (*left*) and within -1Kb of the transcription start site (TSS) and +1Kb of the transcription termination site (TTS) of rhythmic genes differentially regulated in *Clk* knockouts (*right*) in all genotypes at ZT04 and at ZT16.
(TIF)
