# Supplementary material for: Genome-wide discovery of the daily transcriptome, DNA regulatory elements and transcription factor occupancy in the monarch butterfly brain
Source: PLoS Genet. 2019 Jul 23;15(7):e1008265. doi: 10.1371/journal.pgen.1008265 (PMC6677324; doi:10.1371/journal.pgen.1008265)
Supplement: S1 Table — (DOCX) [file pgen.1008265.s001.docx]

**S1 Table.** Rhythmic genes identified by either RAIN or MetaCycle with adjusted *p*-value (adjP) ≤ 0.05 and fold-change (maximum/minimum expression values) ≥ 1.3.

| **geneID** | **symbol** | **name** | **adjP** | **Fold-change** |
| --- | --- | --- | --- | --- |
| DPOGS204644 | CG11438 | CG11438 | 5.00E-09 | 8.1 |
| DPOGS211121 | CG4502 | CG4502 | 1.72E-08 | 2.2 |
| DPOGS205549 | CG43795 | CG43795 | 2.72E-08 | 2 |
| DPOGS207730 | CG10082 | CG10082 | 2.09E-07 | 2.9 |
| DPOGS210128 | Papss | PAPS synthetase | 2.09E-07 | 1.9 |
| DPOGS213900 | Hsp68 | Heat shock protein 68 | 2.19E-07 | 6.4 |
| DPOGS201544 | Oatp74D | Organic anion transporting polypeptide 74D | 2.19E-07 | 2 |
| DPOGS210295 | Pgi | Phosphoglucose isomerase | 2.19E-07 | 1.6 |
| DPOGS201012 | Lrpprc2 | Leucine-rich pentatricopeptide repeat containing 2 | 2.33E-07 | 3.5 |
| DPOGS214179 | tim | timeless | 5.48E-07 | 3.1 |
| DPOGS214070 | stumps | stumps | 8.55E-07 | 2 |
| DPOGS203908 | per | period | 1.18E-06 | 5.5 |
| DPOGS204494 | GABA-B-R1 | metabotropic GABA-B receptor subtype 1 | 1.27E-06 | 1.5 |
| DPOGS207058 | CG9518 | CG9518 | 1.28E-06 | 10.4 |
| DPOGS207764 | Eno | Enolase | 2.32E-06 | 1.5 |
| DPOGS208959 | Vha100-2 | Vacuolar H[+] ATPase 100kD subunit 2 | 2.32E-06 | 1.5 |
| DPOGS201013 | CG13868 | CG13868 | 2.41E-06 | 3.1 |
| DPOGS209175 | CG14945 | CG14945 | 2.48E-06 | 10.5 |
| DPOGS203088 | CG7720 | CG7720 | 2.79E-06 | 4.9 |
| DPOGS209874 | CG33281 | CG33281 | 2.79E-06 | 4.7 |
| DPOGS215738 | egr | eiger | 2.79E-06 | 2.2 |
| DPOGS208606 | vri | vrille | 2.86E-06 | 5.8 |
| DPOGS207942 | CG10660 | CG10660 | 2.86E-06 | 4.5 |
| DPOGS215160 | Tret1-2 | Trehalose transporter 1-2 | 3.68E-06 | 4.5 |
| DPOGS204253 | CG32032 | CG32032 | 5.81E-06 | 2.3 |
| DPOGS213925 | Hsp68 | Heat shock protein 68 | 7.01E-06 | 6.7 |
| DPOGS213114 | Ctl2 | Choline transporter-like 2 | 9.74E-06 | 2.1 |
| DPOGS200985 | Lgr1 | Leucine-rich repeat-containing G protein-coupled receptor 1 | 1.14E-05 | 2.3 |
| DPOGS211148 | uzip | unzipped | 1.35E-05 | 1.5 |
| DPOGS210627 | CG3940 | CG3940 | 1.54E-05 | 1.6 |
| DPOGS213901 | Hsp68 | Heat shock protein 68 | 1.70E-05 | 7.5 |
| DPOGS212557 | CG7646 | CG7646 | 2.44E-05 | 1.3 |
| DPOGS201668 | sog | short gastrulation | 2.88E-05 | 1.5 |
| DPOGS203797 | Hsf | Heat shock factor | 3.10E-05 | 3.1 |
| DPOGS212590 | ninaB | neither inactivation nor afterpotential B | 3.52E-05 | 2.6 |
| DPOGS205027 | GlyP | Glycogen phosphorylase | 3.52E-05 | 1.3 |
| DPOGS211191 | Zip71B | Zinc/iron regulated transporter-related protein 71B | 4.84E-05 | 1.5 |
| DPOGS208579 | CG30089 | CG30089 | 6.55E-05 | 2 |
| DPOGS201195 | CG5535 | CG5535 | 6.92E-05 | 2 |
| DPOGS203813 | CG3376 | CG3376 | 7.07E-05 | 1.7 |
| DPOGS202178 | Got1 | Glutamate oxaloacetate transaminase 1 | 7.10E-05 | 1.6 |
| DPOGS200691 | E(spl)mbeta-HLH | Enhancer of split mbeta, helix-loop-helix | 1.17E-04 | 5.6 |
| DPOGS206646 | Sik2 | Salt-inducible kinase 2 | 1.18E-04 | 1.6 |
| DPOGS202126 | CG32432 | CG32432 | 1.18E-04 | 1.3 |
| DPOGS215479 | CG5235 | CG5235 | 1.23E-04 | 1.8 |
| DPOGS203810 | Pfk | Phosphofructokinase | 1.23E-04 | 1.5 |
| DPOGS214218 | CG2818 | CG2818 | 1.23E-04 | 1.7 |
| DPOGS206412 | CG9416 | CG9416 | 1.24E-04 | 1.6 |
| DPOGS208998 | CG8745 | CG8745 | 1.43E-04 | 2.1 |
| DPOGS209025 | CG43427 | CG43427 | 1.54E-04 | 1.5 |
| DPOGS206136 | CG32369 | CG32369 | 1.56E-04 | 2.2 |
| DPOGS208079 | Cry2 | Cryptochrome 2 | 1.78E-04 | 1.9 |
| DPOGS215377 | CG9220 | CG9220 | 1.98E-04 | 2.2 |
| DPOGS212605 | CG5853 | CG5853 | 2.01E-04 | 5.2 |
| DPOGS215437 | P5cr-2 | Pyrroline-5-carboxylate reductase-like 2 | 2.01E-04 | 2.1 |
| DPOGS215384 | RIOK2 | RIO kinase 2 | 2.01E-04 | 1.8 |
| DPOGS208231 | CG34458 | CG34458 | 2.34E-04 | 3.5 |
| DPOGS212492 | CG31324 | CG31324 | 2.34E-04 | 2.2 |
| DPOGS207224 | pdgy | pudgy | 2.34E-04 | 1.7 |
| DPOGS201194 | CG5535 | CG5535 | 2.42E-04 | 1.8 |
| DPOGS207000 | Mhcl | Myosin heavy chain-like | 2.57E-04 | 1.6 |
| DPOGS213007 | Cyp18a1 | Cytochrome P450-18a1 | 2.59E-04 | 3.8 |
| DPOGS202237 | CG5001 | CG5001 | 3.10E-04 | 2.1 |
| DPOGS212884 | CG44153 | CG44153 | 3.17E-04 | 1.8 |
| DPOGS202114 | crol | crooked legs | 3.17E-04 | 1.3 |
| DPOGS214215 | Nha1 | Na[+]/H[+] hydrogen antiporter 1 | 3.42E-04 | 3.4 |
| DPOGS202827 | santa-maria | scavenger receptor acting in neural tissue and majority of rhodopsin is absent | 3.49E-04 | 1.6 |
| DPOGS213552 | Eip71CD | Ecdysone-induced protein 28/29kD | 3.54E-04 | 1.8 |
| DPOGS200190 | sima | similar | 3.54E-04 | 1.3 |
| DPOGS207088 | CG7879 | CG7879 | 3.85E-04 | 1.8 |
| DPOGS204552 | CG1213 | CG1213 | 4.25E-04 | 10.2 |
| DPOGS213594 | Gbs-76A | Glycogen binding subunit 76A | 4.83E-04 | 1.8 |
| DPOGS215159 | Tret1-1 | Trehalose transporter 1-1 | 4.99E-04 | 5.2 |
| DPOGS202609 | Ace | Acetylcholine esterase | 5.01E-04 | 1.8 |
| DPOGS205079 | CG7632 | CG7632 | 5.06E-04 | 1.8 |
| DPOGS212022 | klar | klarsicht | 6.01E-04 | 1.4 |
| DPOGS212327 | CG11658 | CG11658 | 7.51E-04 | 1.9 |
| DPOGS204181 | Fit1 | Fermitin 1 | 7.51E-04 | 1.7 |
| DPOGS201345 | CG5958 | CG5958 | 7.62E-04 | 5.2 |
| DPOGS208999 | CG7675 | CG7675 | 8.17E-04 | 3.2 |
| DPOGS210344 | mgl | Megalin | 8.21E-04 | 1.3 |
| DPOGS201488 | CG2765 | CG2765 | 8.44E-04 | 2.1 |
| DPOGS207448 | Best1 | Bestrophin 1 | 8.44E-04 | 1.5 |
| DPOGS214162 | Nha1 | Na[+]/H[+] hydrogen antiporter 1 | 9.35E-04 | 3.4 |
| DPOGS206692 | pdgy | pudgy | 9.46E-04 | 3.8 |
| DPOGS205647 | CG17646 | CG17646 | 9.46E-04 | 1.6 |
| DPOGS215624 | GLS | Glutaminase | 9.46E-04 | 1.5 |
| DPOGS206596 | mgl | Megalin | 1.00E-03 | 1.4 |
| DPOGS210716 | ari-2 | ariadne 2 | 1.02E-03 | 1.7 |
| DPOGS210257 | e | ebony | 1.02E-03 | 1.4 |
| DPOGS215237 | Mid1 | Mid1 | 1.07E-03 | 1.5 |
| DPOGS209585 | CG8176 | CG8176 | 1.10E-03 | 1.3 |
| DPOGS207222 | robl | roadblock | 1.14E-03 | 3.7 |
| DPOGS209797 | Vha100-2 | Vacuolar H[+] ATPase 100kD subunit 2 | 1.29E-03 | 1.9 |
| DPOGS202815 | Eaat2 | Excitatory amino acid transporter 2 | 1.29E-03 | 1.7 |
| DPOGS203808 | a | arc | 1.31E-03 | 1.8 |
| DPOGS200764 | wun | wunen | 1.37E-03 | 5.2 |
| DPOGS201036 | net | net | 1.37E-03 | 3 |
| DPOGS212595 | Tps1 | Trehalose-6-phosphate synthase 1 | 1.37E-03 | 2 |
| DPOGS200490 | Mdh1 | Malate dehydrogenase 1 | 1.37E-03 | 1.9 |
| DPOGS204091 | Sik3 | Salt-inducible kinase 3 | 1.37E-03 | 1.9 |
| DPOGS215969 | Taldo | Transaldolase | 1.37E-03 | 1.9 |
| DPOGS212482 | gem | gemini | 1.37E-03 | 1.8 |
| DPOGS207157 | Fhos | Formin homology 2 domain containing | 1.37E-03 | 1.7 |
| DPOGS200407 | muc | midline uncoordinated | 1.37E-03 | 1.4 |
| DPOGS205291 | sxc | super sex combs | 1.37E-03 | 1.3 |
| DPOGS214408 | CG42269 | CG42269 | 1.37E-03 | 2.1 |
| DPOGS204547 | Liprin-gamma | Liprin-gamma | 1.39E-03 | 1.8 |
| DPOGS209507 | SamDC | S-adenosylmethionine decarboxylase | 1.41E-03 | 1.4 |
| DPOGS200817 | CG5150 | CG5150 | 1.56E-03 | 1.7 |
| DPOGS202607 | mamo | maternal gene required for meiosis | 1.56E-03 | 1.5 |
| DPOGS202145 | DIP-gamma | Dpr-interacting protein gamma | 1.57E-03 | 1.4 |
| DPOGS213355 | CG6472 | CG6472 | 1.58E-03 | 7.7 |
| DPOGS201881 | nrv1 | nervana 1 | 1.68E-03 | 2.1 |
| DPOGS205823 | CG33791 | CG33791 | 1.68E-03 | 1.8 |
| DPOGS213793 | v | vermilion | 1.68E-03 | 1.7 |
| DPOGS204626 | CG7582 | CG7582 | 1.68E-03 | 1.5 |
| DPOGS202993 | CG7888 | CG7888 | 1.70E-03 | 1.6 |
| DPOGS208537 | frtz | fritz | 2.04E-03 | 1.5 |
| DPOGS215494 | AGBE | 1,4-Alpha-Glucan Branching Enzyme | 2.11E-03 | 1.6 |
| DPOGS200811 | h | hairy | 2.38E-03 | 3.6 |
| DPOGS208406 | CG30069 | CG30069 | 2.38E-03 | 1.6 |
| DPOGS209166 | Bre1 | Bre1 | 2.44E-03 | 4.9 |
| DPOGS201446 | CG10527 | CG10527 | 2.44E-03 | 2 |
| DPOGS209910 | Sox14 | Sox box protein 14 | 2.48E-03 | 3.3 |
| DPOGS207651 | CG17323 | CG17323 | 2.51E-03 | 3.1 |
| DPOGS210248 | CG42235 | CG42235 | 2.67E-03 | 4.2 |
| DPOGS204642 | Graf | GTPase regulator associated with FAK | 2.67E-03 | 2.1 |
| DPOGS213327 | CG7110 | CG7110 | 2.67E-03 | 1.7 |
| DPOGS211699 | CG3328 | CG3328 | 2.67E-03 | 1.6 |
| DPOGS207077 | CG13366 | CG13366 | 2.72E-03 | 1.5 |
| DPOGS213064 | Pgk | Phosphoglycerate kinase | 2.78E-03 | 2 |
| DPOGS211900 | aop | anterior open | 3.09E-03 | 1.8 |
| DPOGS214921 | Mco1 | Multicopper oxidase-1 | 3.09E-03 | 1.5 |
| DPOGS214402 | GlyS | Glycogen synthase | 3.24E-03 | 1.5 |
| DPOGS208881 | Droj2 | DnaJ-like-2 | 3.44E-03 | 1.6 |
| DPOGS215458 | CG10428 | CG10428 | 3.55E-03 | 2.4 |
| DPOGS213546 | CG10089 | CG10089 | 3.55E-03 | 2.1 |
| DPOGS214262 | CG9932 | CG9932 | 3.60E-03 | 2 |
| DPOGS205927 | spen | split ends | 3.60E-03 | 1.8 |
| DPOGS203887 | Invadolysin | Invadolysin | 3.60E-03 | 1.7 |
| DPOGS207003 | gd | gastrulation-defective | 3.70E-03 | 13.9 |
| DPOGS215439 | l(2)efl | lethal (2) essential for life | 3.77E-03 | 9.5 |
| DPOGS214745 | CG9413 | CG9413 | 3.77E-03 | 1.6 |
| DPOGS200089 | Tpi | Triose phosphate isomerase | 3.91E-03 | 2.1 |
| DPOGS202434 | e | ebony | 3.91E-03 | 2.5 |
| DPOGS212889 | CG1218 | CG1218 | 4.02E-03 | 16.4 |
| DPOGS204153 | Neurochondrin | Neurochondrin | 4.09E-03 | 2.5 |
| DPOGS215488 | CG7376 | CG7376 | 4.12E-03 | 1.8 |
| DPOGS212996 | ImpE1 | Ecdysone-inducible gene E1 | 4.33E-03 | 2.5 |
| DPOGS212858 | atl | atlastin | 4.37E-03 | 1.5 |
| DPOGS202781 | Hop | Hsp70/Hsp90 organizing protein | 5.02E-03 | 1.5 |
| DPOGS215981 | SREBP | Sterol regulatory element binding protein | 5.11E-03 | 1.6 |
| DPOGS214045 | CG4341 | CG4341 | 5.11E-03 | 1.4 |
| DPOGS215460 | Gapdh2 | Glyceraldehyde 3 phosphate dehydrogenase 2 | 5.11E-03 | 1.3 |
| DPOGS204998 | Ccp84Ae | Ccp84Ae | 5.13E-03 | 6 |
| DPOGS215489 | Pfrx | 6-phosphofructo-2-kinase | 5.13E-03 | 2.1 |
| DPOGS205557 | Cenp-C | Centromeric protein-C | 5.13E-03 | 2.1 |
| DPOGS213207 | CG1667 | CG1667 | 5.13E-03 | 1.8 |
| DPOGS200883 | CG16791 | CG16791 | 5.13E-03 | 1.6 |
| DPOGS205152 | ImpL2 | Ecdysone-inducible gene L2 | 5.23E-03 | 8.1 |
| DPOGS201845 | wb | wing blister | 5.25E-03 | 1.6 |
| DPOGS203228 | Cln7 | Cln7 | 5.53E-03 | 2 |
| DPOGS201463 | dve | defective proventriculus | 5.86E-03 | 1.6 |
| DPOGS216202 | cas | castor | 6.09E-03 | 1.3 |
| DPOGS212748 | sea | scheggia | 6.46E-03 | 1.6 |
| DPOGS200356 | HEATR2 | HEAT repeat containing 2 | 7.07E-03 | 1.8 |
| DPOGS211388 | CG2082 | CG2082 | 7.18E-03 | 2.7 |
| DPOGS201573 | cbt | cabut | 7.19E-03 | 3.3 |
| DPOGS210492 | Cpsf73 | Cleavage and polyadenylation specificity factor 73 | 7.19E-03 | 2.6 |
| DPOGS201241 | CAP | CAP | 7.19E-03 | 2.5 |
| DPOGS206850 | CG7518 | CG7518 | 7.19E-03 | 2.1 |
| DPOGS205582 | CG6178 | CG6178 | 7.19E-03 | 1.6 |
| DPOGS208596 | CG15186 | CG15186 | 7.19E-03 | 1.4 |
| DPOGS207480 | Ilp3 | Insulin-like peptide 3 | 7.23E-03 | 2.8 |
| DPOGS213804 | IP3K1 | Inositol 1,4,5-triphosphate kinase 1 | 7.23E-03 | 2.7 |
| DPOGS212829 | CG10175 | CG10175 | 7.23E-03 | 1.8 |
| DPOGS213444 | KdelR | KDEL receptor | 7.23E-03 | 1.8 |
| DPOGS214597 | TrpRS | Tryptophanyl-tRNA synthetase | 7.23E-03 | 1.3 |
| DPOGS204363 | kek2 | kekkon-2 | 7.24E-03 | 1.5 |
| DPOGS205706 | CG42588 | CG42588 | 7.42E-03 | 4.2 |
| DPOGS208868 | Membrin | Membrin | 7.42E-03 | 2.4 |
| DPOGS214099 | CG3792 | CG3792 | 7.53E-03 | 3.6 |
| DPOGS209864 | jim | jim | 7.96E-03 | 1.3 |
| DPOGS205395 | NA | No annotation | 7.98E-03 | 5 |
| DPOGS204463 | Mmp1 | Matrix metalloproteinase 1 | 8.01E-03 | 7.4 |
| DPOGS206156 | CG15919 | CG15919 | 8.17E-03 | 6.1 |
| DPOGS203710 | CG8602 | CG8602 | 8.69E-03 | 1.9 |
| DPOGS205105 | CG4822 | CG4822 | 9.39E-03 | 2.8 |
| DPOGS211169 | mRpL3 | mitochondrial ribosomal protein L3 | 9.44E-03 | 2.3 |
| DPOGS213302 | sinah | sina homologue | 9.79E-03 | 3.7 |
| DPOGS202408 | dlp | dally-like | 9.79E-03 | 3.1 |
| DPOGS204024 | Mocs2 | Molybdenum cofactor synthesis 2 | 9.79E-03 | 2.1 |
| DPOGS210992 | Mtl | Mig-2-like | 9.84E-03 | 1.6 |
| DPOGS212101 | fabp | fatty acid binding protein | 9.91E-03 | 8 |
| DPOGS207444 | CG42237 | CG42237 | 9.91E-03 | 2.2 |
| DPOGS212406 | Exn | Ephexin | 9.91E-03 | 2.2 |
| DPOGS206382 | l(2)efl | lethal (2) essential for life | 9.97E-03 | 6.7 |
| DPOGS215419 | dpy | dumpy | 9.97E-03 | 2.4 |
| DPOGS205824 | pigs | pickled eggs | 9.97E-03 | 2 |
| DPOGS209035 | CG8036 | CG8036 | 9.97E-03 | 1.9 |
| DPOGS213854 | vlc | vulcan | 1.06E-02 | 1.4 |
| DPOGS212685 | CBP | sarcoplasmic calcium-binding protein | 1.06E-02 | 2.4 |
| DPOGS201651 | NC2alpha | Negative Cofactor 2alpha | 1.06E-02 | 1.8 |
| DPOGS208883 | Gpdh | Glycerol 3 phosphate dehydrogenase | 1.08E-02 | 1.7 |
| DPOGS206718 | CG32564 | CG32564 | 1.09E-02 | 4.8 |
| DPOGS201162 | Esp | Epidermal stripes and patches | 1.10E-02 | 1.8 |
| DPOGS214879 | pn | prune | 1.10E-02 | 2.1 |
| DPOGS203868 | CG43693 | CG43693 | 1.16E-02 | 2 |
| DPOGS213326 | CG7110 | CG7110 | 1.16E-02 | 1.5 |
| DPOGS201376 | Neurl4 | Neuralized E3 ubiquitin protein ligase 4 | 1.17E-02 | 1.4 |
| DPOGS212989 | CG10904 | CG10904 | 1.18E-02 | 1.7 |
| DPOGS205911 | Vps20 | Vacuolar protein sorting 20 | 1.19E-02 | 4.1 |
| DPOGS204296 | Parg | Poly(ADP-ribose) glycohydrolase | 1.23E-02 | 2.3 |
| DPOGS201894 | Mal-A4 | Maltase A4 | 1.27E-02 | 2.1 |
| DPOGS207405 | Pcf11 | Protein 1 of cleavage and polyadenylation factor 1 | 1.27E-02 | 1.4 |
| DPOGS215481 | nompC | no mechanoreceptor potential C | 1.28E-02 | 16.8 |
| DPOGS213502 | Fdx1 | Ferredoxin 1 | 1.28E-02 | 4.5 |
| DPOGS204673 | CG13813 | CG13813 | 1.28E-02 | 2.7 |
| DPOGS207391 | CG10237 | CG10237 | 1.28E-02 | 2.7 |
| DPOGS208044 | NKCC | sodium potassium chloride cotransporter | 1.28E-02 | 2.4 |
| DPOGS209609 | Madm | MLF1-adaptor molecule | 1.28E-02 | 2 |
| DPOGS211461 | CG8312 | CG8312 | 1.28E-02 | 1.8 |
| DPOGS214041 | beta-Spec | beta Spectrin | 1.28E-02 | 1.7 |
| DPOGS203923 | l(1)G0289 | lethal (1) G0289 | 1.28E-02 | 1.7 |
| DPOGS202879 | Nup44A | Nucleoporin at 44A | 1.28E-02 | 1.5 |
| DPOGS202283 | CG1646 | CG1646 | 1.28E-02 | 1.4 |
| DPOGS211367 | CoRest | CoRest | 1.28E-02 | 1.4 |
| DPOGS205373 | Fer2LCH | Ferritin 2 light chain homologue | 1.28E-02 | 1.4 |
| DPOGS205265 | CG13315 | CG13315 | 1.28E-02 | 2.2 |
| DPOGS200426 | CG30460 | CG30460 | 1.28E-02 | 1.6 |
| DPOGS203920 | CG7888 | CG7888 | 1.29E-02 | 1.5 |
| DPOGS210801 | wake | wide awake | 1.30E-02 | 1.8 |
| DPOGS213632 | pall | pallbearer | 1.34E-02 | 1.9 |
| DPOGS203791 | Sema1b | Semaphorin 1b | 1.34E-02 | 1.6 |
| DPOGS215591 | CG10431 | CG10431 | 1.38E-02 | 2 |
| DPOGS203890 | santa-maria | scavenger receptor acting in neural tissue and majority of rhodopsin is absent | 1.43E-02 | 1.5 |
| DPOGS208188 | Sema5c | Semaphorin 5c | 1.44E-02 | 1.9 |
| DPOGS212885 | Kdm2 | Lysine (K)-specific demethylase 2 | 1.44E-02 | 2.2 |
| DPOGS213701 | me31B | maternal expression at 31B | 1.46E-02 | 1.4 |
| DPOGS211474 | pst | pastrel | 1.51E-02 | 1.5 |
| DPOGS212608 | CG11601 | CG11601 | 1.51E-02 | 1.7 |
| DPOGS201726 | CG5493 | CG5493 | 1.55E-02 | 2.2 |
| DPOGS212145 | AdoR | Adenosine receptor | 1.59E-02 | 1.7 |
| DPOGS215783 | hook | hook | 1.67E-02 | 1.6 |
| DPOGS211138 | Rgl | Ral guanine nucleotide dissociation stimulator-like | 1.74E-02 | 1.8 |
| DPOGS210186 | Pdk | Pyruvate dehydrogenase kinase | 1.76E-02 | 1.7 |
| DPOGS214570 | MP1 | Melanization Protease 1 | 1.78E-02 | 4.9 |
| DPOGS213690 | beat-IIIc | beat-IIIc | 1.78E-02 | 3.3 |
| DPOGS205833 | CG9485 | CG9485 | 1.78E-02 | 2.1 |
| DPOGS207621 | Lrch | Leucine-rich-repeats and calponin homology domain protein | 1.81E-02 | 15.2 |
| DPOGS208120 | CG8545 | CG8545 | 1.81E-02 | 2.1 |
| DPOGS209167 | Ssadh | Succinic semialdehyde dehydrogenase | 1.81E-02 | 1.7 |
| DPOGS204377 | CG9171 | CG9171 | 1.81E-02 | 1.4 |
| DPOGS214709 | CG14683 | CG14683 | 1.85E-02 | 3.1 |
| DPOGS214927 | Sin3A | Sin3A | 1.85E-02 | 1.7 |
| DPOGS208609 | Adf1 | Adh transcription factor 1 | 1.85E-02 | 1.4 |
| DPOGS215242 | chico | chico | 1.90E-02 | 1.9 |
| DPOGS214042 | beta-Spec | beta Spectrin | 1.91E-02 | 1.7 |
| DPOGS202467 | CG4022 | CG4022 | 1.91E-02 | 1.6 |
| DPOGS204466 | IntS8 | Integrator 8 | 1.92E-02 | 1.3 |
| DPOGS214306 | CG10602 | CG10602 | 1.95E-02 | 2 |
| DPOGS212844 | Ctr1A | Copper transporter 1A | 1.95E-02 | 1.9 |
| DPOGS213986 | CG5033 | CG5033 | 1.95E-02 | 1.5 |
| DPOGS215868 | CG9328 | CG9328 | 1.99E-02 | 1.5 |
| DPOGS213560 | nuf | nuclear fallout | 2.03E-02 | 1.7 |
| DPOGS209834 | CG14516 | CG14516 | 2.04E-02 | 1.7 |
| DPOGS208578 | VGlut | Vesicular glutamate transporter | 2.15E-02 | 1.3 |
| DPOGS212481 | gem | gemini | 2.20E-02 | 1.5 |
| DPOGS200138 | Nsun5 | Nop2/Sun-like domain containing protein 5 | 2.21E-02 | 1.8 |
| DPOGS208306 | Ance-3 | Ance-3 | 2.21E-02 | 7.3 |
| DPOGS202245 | Sras | severas | 2.21E-02 | 3.8 |
| DPOGS207274 | Socs16D | Suppressor of Cytokine Signaling at 16D | 2.21E-02 | 3.3 |
| DPOGS215104 | Gbs-70E | Glycogen binding subunit 70E | 2.21E-02 | 2.7 |
| DPOGS211389 | CG2082 | CG2082 | 2.21E-02 | 2.2 |
| DPOGS202655 | Cyt-b5 | Cytochrome b5 | 2.21E-02 | 2.1 |
| DPOGS213282 | zld | zelda | 2.21E-02 | 1.6 |
| DPOGS200872 | RpL27 | Ribosomal protein L27 | 2.21E-02 | 1.5 |
| DPOGS214212 | c12.1 | c12.1 | 2.21E-02 | 1.4 |
| DPOGS213384 | RpS20 | Ribosomal protein S20 | 2.21E-02 | 1.4 |
| DPOGS214111 | Glg1 | Golgi complex-localized glycoprotein 1 | 2.25E-02 | 2.5 |
| DPOGS211174 | Cwc25 | Cwc25 | 2.25E-02 | 1.7 |
| DPOGS215720 | Argk | Arginine kinase | 2.25E-02 | 1.5 |
| DPOGS210777 | CG2991 | CG2991 | 2.25E-02 | 1.5 |
| DPOGS204620 | Su(fu) | Suppressor of fused | 2.27E-02 | 2 |
| DPOGS203555 | Odc1 | Ornithine decarboxylase 1 | 2.27E-02 | 1.9 |
| DPOGS213878 | CG3168 | CG3168 | 2.27E-02 | 1.8 |
| DPOGS205677 | Sap30 | SIN3-associated polypeptide 30 | 2.27E-02 | 1.7 |
| DPOGS206078 | CG5687 | CG5687 | 2.27E-02 | 1.6 |
| DPOGS201954 | Usp2 | Ubiquitin specific protease 2 | 2.27E-02 | 1.5 |
| DPOGS216046 | Nplp1 | Neuropeptide-like precursor 1 | 2.27E-02 | 1.4 |
| DPOGS214351 | Hil | Hillarin | 2.32E-02 | 2.1 |
| DPOGS212365 | Prosalpha1 | Proteasome alpha1 subunit | 2.32E-02 | 1.3 |
| DPOGS206473 | CG4797 | CG4797 | 2.38E-02 | 1.5 |
| DPOGS205442 | ktub | king tubby | 2.39E-02 | 1.5 |
| DPOGS206893 | Snap29 | Synaptosomal-associated protein 29kDa | 2.42E-02 | 1.7 |
| DPOGS200737 | LeuRS | Leucyl-tRNA synthetase | 2.42E-02 | 1.3 |
| DPOGS209352 | RpL38 | Ribosomal protein L38 | 2.43E-02 | 1.4 |
| DPOGS212943 | Rgk3 | Rad, Gem/Kir family member 3 | 2.48E-02 | 4.4 |
| DPOGS205353 | Mys45A | Mystery 45A | 2.48E-02 | 1.8 |
| DPOGS213168 | tkv | thickveins | 2.62E-02 | 3.5 |
| DPOGS201422 | CG5009 | CG5009 | 2.62E-02 | 1.6 |
| DPOGS202263 | CG3756 | CG3756 | 2.64E-02 | 1.9 |
| DPOGS215318 | ssp7 | short spindle 7 | 2.69E-02 | 8.9 |
| DPOGS211581 | scrib | scribbled | 2.76E-02 | 1.5 |
| DPOGS205649 | CG5853 | CG5853 | 2.77E-02 | 1.9 |
| DPOGS213576 | Hexo2 | Hexosaminidase 2 | 2.89E-02 | 2.7 |
| DPOGS206428 | galla-1 | galla-1 | 2.89E-02 | 1.8 |
| DPOGS215265 | CG13398 | CG13398 | 2.94E-02 | 1.4 |
| DPOGS209925 | cwo | clockwork orange | 2.98E-02 | 5.3 |
| DPOGS200824 | CG13124 | CG13124 | 3.02E-02 | 1.9 |
| DPOGS205613 | CG3735 | CG3735 | 3.05E-02 | 1.8 |
| DPOGS200281 | isoQC | iso Glutaminyl cyclase | 3.05E-02 | 2.5 |
| DPOGS202602 | Mcm2 | Minichromosome maintenance 2 | 3.06E-02 | 2.2 |
| DPOGS201445 | CG14984 | CG14984 | 3.07E-02 | 2.2 |
| DPOGS205239 | VhaAC45 | Vacuolar H[+] ATPase AC45 accessory subunit | 3.07E-02 | 1.3 |
| DPOGS206043 | ftz-f1 | ftz transcription factor 1 | 3.10E-02 | 1.9 |
| DPOGS213288 | Nab2 | Nuclear polyadenosine RNA-binding 2 | 3.10E-02 | 1.7 |
| DPOGS200168 | Tsp29Fa | Tetraspanin 29Fa | 3.11E-02 | 14 |
| DPOGS208217 | Unc-115a | Uncoordinated 115a | 3.11E-02 | 2.4 |
| DPOGS214481 | Wnk | Wnk kinase | 3.11E-02 | 2 |
| DPOGS202068 | Ric | Ras-related protein interacting with calmodulin | 3.12E-02 | 4.5 |
| DPOGS205855 | CG15890 | CG15890 | 3.12E-02 | 2.7 |
| DPOGS207714 | aay | astray | 3.12E-02 | 2.2 |
| DPOGS206135 | Mdr65 | Multi drug resistance 65 | 3.12E-02 | 2.1 |
| DPOGS213666 | Cpr49Ag | Cuticular protein 49Ag | 3.12E-02 | 2 |
| DPOGS210731 | NA | No annotation | 3.12E-02 | 2 |
| DPOGS209948 | regucalcin | regucalcin | 3.12E-02 | 1.7 |
| DPOGS206959 | Ald | Aldolase | 3.12E-02 | 1.5 |
| DPOGS216184 | l(2)efl | lethal (2) essential for life | 3.12E-02 | 1.5 |
| DPOGS204292 | Sulf1 | Sulfated | 3.12E-02 | 1.4 |
| DPOGS207212 | CG7550 | CG7550 | 3.40E-02 | 1.6 |
| DPOGS201813 | Pglym78 | Phosphoglyceromutase | 3.41E-02 | 1.6 |
| DPOGS212385 | l(1)G0007 | lethal (1) G0007 | 3.50E-02 | 1.9 |
| DPOGS202608 | mamo | maternal gene required for meiosis | 3.50E-02 | 1.7 |
| DPOGS210087 | unc-5 | unc-5 | 3.51E-02 | 1.4 |
| DPOGS203552 | CG18155 | CG18155 | 3.52E-02 | 2 |
| DPOGS214482 | NA | No annotation | 3.54E-02 | 2.4 |
| DPOGS202825 | fusl | fuseless | 3.54E-02 | 1.5 |
| DPOGS202204 | OstDelta | Oligosaccharide transferase Delta subunit | 3.54E-02 | 1.3 |
| DPOGS210286 | Dek | Dek | 3.60E-02 | 8.2 |
| DPOGS209186 | Cad86C | Cadherin 86C | 3.61E-02 | 1.7 |
| DPOGS200039 | Ufc1 | Ubiquitin-fold modifier conjugating enzyme 1 | 3.63E-02 | 1.6 |
| DPOGS203970 | DUBAI | Deubiquitinating apoptotic inhibitor | 3.63E-02 | 1.4 |
| DPOGS215699 | CG2906 | CG2906 | 3.63E-02 | 3.6 |
| DPOGS201763 | RagC-D | Ras-related GTP binding C/D | 3.63E-02 | 3.2 |
| DPOGS206457 | comm3 | comm3 | 3.63E-02 | 2.4 |
| DPOGS208760 | 5-HT2B | 5-hydroxytryptamine (serotonin) receptor 2B | 3.63E-02 | 2.2 |
| DPOGS206924 | bi | bifid | 3.63E-02 | 2.2 |
| DPOGS209521 | Faa | Fumarylacetoacetase | 3.63E-02 | 1.9 |
| DPOGS207451 | GlnRS | Glutaminyl-tRNA synthetase | 3.63E-02 | 1.9 |
| DPOGS212948 | CG5059 | CG5059 | 3.63E-02 | 1.8 |
| DPOGS212266 | CG13907 | CG13907 | 3.63E-02 | 1.5 |
| DPOGS202648 | Pld | Phospholipase D | 3.63E-02 | 1.5 |
| DPOGS206348 | Picot | Picot | 3.63E-02 | 1.4 |
| DPOGS213877 | Dad | Daughters against dpp | 3.63E-02 | 2.9 |
| DPOGS213057 | Drip | Drip | 3.63E-02 | 2.4 |
| DPOGS215784 | nw | narrow | 3.63E-02 | 2.1 |
| DPOGS210302 | Myb | Myb oncogene-like | 3.63E-02 | 1.5 |
| DPOGS200016 | Nlg3 | Neuroligin 3 | 3.63E-02 | 1.5 |
| DPOGS203996 | RhoGAP15B | Rho GTPase activating protein at 15B | 3.63E-02 | 1.5 |
| DPOGS202137 | Crag | Calmodulin-binding protein related to a Rab3 GDP/GTP exchange protein | 3.63E-02 | 1.4 |
| DPOGS212305 | kek6 | kek6 | 3.66E-02 | 1.9 |
| DPOGS208185 | Scsalpha | Succinyl coenzyme A synthetase alpha subunit | 3.66E-02 | 1.3 |
| DPOGS207354 | pyd | polychaetoid | 3.66E-02 | 3 |
| DPOGS215967 | Mob2 | Mob2 | 3.66E-02 | 2.1 |
| DPOGS202101 | fng | fringe | 3.66E-02 | 1.9 |
| DPOGS201743 | Lpin | Lipin | 3.66E-02 | 1.6 |
| DPOGS207338 | CG40160 | CG40160 | 3.66E-02 | 1.4 |
| DPOGS203049 | BI-1 | Bax Inhibitor-1 | 3.66E-02 | 1.4 |
| DPOGS207382 | VhaSFD | Vacuolar H[+]-ATPase SFD subunit | 3.66E-02 | 1.3 |
| DPOGS209979 | NA | No annotation | 3.68E-02 | 16.5 |
| DPOGS202823 | CG11159 | CG11159 | 3.68E-02 | 11.4 |
| DPOGS204461 | ST6Gal | Sialyltransferase | 3.68E-02 | 2.4 |
| DPOGS214925 | mRpL45 | mitochondrial ribosomal protein L45 | 3.68E-02 | 2.3 |
| DPOGS213913 | CG4797 | CG4797 | 3.68E-02 | 2.2 |
| DPOGS201874 | CG13284 | CG13284 | 3.68E-02 | 2.1 |
| DPOGS203882 | mahj | mahjong | 3.68E-02 | 2.1 |
| DPOGS211220 | Ddc | Dopa decarboxylase | 3.68E-02 | 1.8 |
| DPOGS211089 | jp | junctophilin | 3.68E-02 | 1.8 |
| DPOGS213859 | dgt1 | dim gamma-tubulin 1 | 3.68E-02 | 1.6 |
| DPOGS213275 | CG1504 | CG1504 | 3.68E-02 | 1.3 |
| DPOGS203181 | CG3829 | CG3829 | 3.75E-02 | 3.8 |
| DPOGS213717 | CG9018 | CG9018 | 3.75E-02 | 2.1 |
| DPOGS205038 | CG42668 | CG42668 | 3.75E-02 | 1.8 |
| DPOGS202213 | CG6443 | CG6443 | 3.75E-02 | 1.5 |
| DPOGS209591 | osp | outspread | 3.75E-02 | 1.5 |
| DPOGS215947 | CG14906 | CG14906 | 3.80E-02 | 5.5 |
| DPOGS205076 | CG12484 | CG12484 | 3.82E-02 | 1.6 |
| DPOGS211800 | CG31156 | CG31156 | 3.82E-02 | 1.6 |
| DPOGS203515 | MED25 | Mediator complex subunit 25 | 3.82E-02 | 1.6 |
| DPOGS200739 | Tdrd3 | Tudor domain containing 3 | 3.82E-02 | 1.5 |
| DPOGS212976 | Pdh | Photoreceptor dehydrogenase | 3.82E-02 | 1.3 |
| DPOGS210848 | stv | starvin | 3.87E-02 | 1.4 |
| DPOGS202573 | Pepck | Phosphoenolpyruvate carboxykinase | 3.91E-02 | 30.4 |
| DPOGS207355 | pyd | polychaetoid | 3.91E-02 | 1.4 |
| DPOGS215527 | CG14814 | CG14814 | 3.92E-02 | 1.8 |
| DPOGS207445 | CG16979 | CG16979 | 3.94E-02 | 2.2 |
| DPOGS215611 | Rpn13 | Regulatory particle non-ATPase 13 | 3.99E-02 | 1.4 |
| DPOGS206538 | CG10621 | CG10621 | 4.05E-02 | 1.7 |
| DPOGS213522 | CenB1A | Centaurin beta 1A | 4.08E-02 | 7.4 |
| DPOGS203264 | sle | slender lobes | 4.08E-02 | 1.8 |
| DPOGS203155 | Cul5 | Cullin 5 | 4.10E-02 | 1.6 |
| DPOGS200617 | RunxB | Runt related B | 4.15E-02 | 7.3 |
| DPOGS214559 | crol | crooked legs | 4.15E-02 | 1.5 |
| DPOGS207057 | CG45065 | CG45065 | 4.34E-02 | 3.5 |
| DPOGS207871 | Grip163 | Grip163 | 4.34E-02 | 1.6 |
| DPOGS214349 | wun | wunen | 4.34E-02 | 3.6 |
| DPOGS210680 | Kal1 | Kallmann syndrome 1 | 4.36E-02 | 1.5 |
| DPOGS201429 | cac | cacophony | 4.49E-02 | 5.8 |
| DPOGS203015 | CG4554 | CG4554 | 4.69E-02 | 16.8 |
| DPOGS205964 | Usp20-33 | Ubiquitin specific protease 20/33 | 4.69E-02 | 3.1 |
| DPOGS209183 | sev | sevenless | 4.69E-02 | 1.3 |
| DPOGS209508 | Pgm | phosphoglucose mutase | 4.73E-02 | 1.7 |
| DPOGS212307 | Tbc1d15-17 | TBC1 domain family member 15/17 | 4.76E-02 | 6.1 |
| DPOGS209143 | CG7458 | CG7458 | 4.82E-02 | 2.1 |
| DPOGS215016 | CG7372 | CG7372 | 4.86E-02 | 1.7 |
| DPOGS205264 | CG7470 | CG7470 | 4.86E-02 | 2.2 |
| DPOGS200409 | Itgbn | Integrin betanu subunit | 4.86E-02 | 2 |
| DPOGS200157 | Rab5 | Rab5 | 4.86E-02 | 1.8 |
| DPOGS206638 | Hers | Histone gene-specific Epigenetic Repressor in late S phase | 4.86E-02 | 1.6 |
| DPOGS210059 | Tet | Ten-Eleven Translocation (TET) family protein | 4.86E-02 | 1.3 |
| DPOGS208916 | CG16789 | CG16789 | 4.90E-02 | 5.6 |
| DPOGS208405 | CG30069 | CG30069 | 4.90E-02 | 1.6 |
| DPOGS216126 | Gcn5 | Gcn5 acetyltransferase | 4.90E-02 | 2.4 |
| DPOGS209271 | CG9896 | CG9896 | 4.93E-02 | 2.7 |
| DPOGS208171 | CG7878 | CG7878 | 4.93E-02 | 1.9 |
| DPOGS213508 | CG6700 | CG6700 | 4.93E-02 | 1.5 |
| DPOGS203775 | NfI | Nuclear factor I | 4.93E-02 | 1.5 |
| DPOGS202256 | Rpt2 | Regulatory particle triple-A ATPase 2 | 4.93E-02 | 1.4 |
| DPOGS211888 | CG42265 | CG42265 | 4.98E-02 | 1.3 |
