# Supplementary material for: Genome-wide discovery of the daily transcriptome, DNA regulatory elements and transcription factor occupancy in the monarch butterfly brain
Source: PLoS Genet. 2019 Jul 23;15(7):e1008265. doi: 10.1371/journal.pgen.1008265 (PMC6677324; doi:10.1371/journal.pgen.1008265)
Supplement: S3 Table — (DOCX) [file pgen.1008265.s003.docx]

**S3 Table.** Rhythmic genes in wild-type differentially expressed in *Cry2* knockouts with adjusted *p*-value (adjP) ≤ 0.05 from robust DODR method.

| **geneID** | **symbol** | **name** | **adjP** |
| --- | --- | --- | --- |
| DPOGS207000 | Mhcl | Myosin heavy chain-like | 5.52E-04 |
| DPOGS203908 | per | period | 5.52E-04 |
| DPOGS213900 | Hsp68 | Heat shock protein 68 | 9.64E-04 |
| DPOGS214179 | tim | timeless | 9.64E-04 |
| DPOGS210128 | Papss | PAPS synthetase | 9.67E-04 |
| DPOGS204644 | CG11438 | CG11438 | 9.67E-04 |
| DPOGS207058 | CG9518 | CG9518 | 1.39E-03 |
| DPOGS211121 | CG4502 | CG4502 | 1.60E-03 |
| DPOGS213552 | Eip71CD | Ecdysone-induced protein 28/29kD | 1.86E-03 |
| DPOGS213901 | Hsp68 | Heat shock protein 68 | 1.97E-03 |
| DPOGS204253 | CG32032 | CG32032 | 2.03E-03 |
| DPOGS205549 | CG43795 | CG43795 | 2.09E-03 |
| DPOGS207730 | CG10082 | CG10082 | 2.93E-03 |
| DPOGS213925 | Hsp68 | Heat shock protein 68 | 2.93E-03 |
| DPOGS203797 | Hsf | Heat shock factor | 2.93E-03 |
| DPOGS208959 | Vha100-2 | Vacuolar H[+] ATPase 100kD subunit 2 | 2.93E-03 |
| DPOGS211474 | pst | pastrel | 3.09E-03 |
| DPOGS210257 | e | ebony | 3.31E-03 |
| DPOGS201544 | Oatp74D | Organic anion transporting polypeptide 74D | 3.62E-03 |
| DPOGS200490 | Mdh1 | Malate dehydrogenase 1 | 3.67E-03 |
| DPOGS201881 | nrv1 | nervana 1 | 4.02E-03 |
| DPOGS201012 | Lrpprc2 | Leucine-rich pentatricopeptide repeat containing 2 | 4.60E-03 |
| DPOGS208606 | vri | vrille | 5.04E-03 |
| DPOGS211169 | mRpL3 | mitochondrial ribosomal protein L3 | 5.22E-03 |
| DPOGS209521 | Faa | Fumarylacetoacetase | 5.22E-03 |
| DPOGS201195 | CG5535 | CG5535 | 5.91E-03 |
| DPOGS213064 | Pgk | Phosphoglycerate kinase | 6.18E-03 |
| DPOGS215419 | dpy | dumpy | 6.18E-03 |
| DPOGS208079 | Cry2 | Cryptochrome 2 | 6.40E-03 |
| DPOGS200691 | E(spl)mbeta-HLH | Enhancer of split mbeta, helix-loop-helix | 8.00E-03 |
| DPOGS203920 | CG7888 | CG7888 | 8.00E-03 |
| DPOGS202655 | Cyt-b5 | Cytochrome b5 | 8.00E-03 |
| DPOGS212608 | CG11601 | CG11601 | 8.34E-03 |
| DPOGS202609 | Ace | Acetylcholine esterase | 8.34E-03 |
| DPOGS203810 | Pfk | Phosphofructokinase | 8.73E-03 |
| DPOGS201013 | CG13868 | CG13868 | 1.09E-02 |
| DPOGS202145 | DIP-gamma | Dpr-interacting protein gamma | 1.09E-02 |
| DPOGS213114 | Ctl2 | Choline transporter-like 2 | 1.12E-02 |
| DPOGS203088 | CG7720 | CG7720 | 1.14E-02 |
| DPOGS215489 | Pfrx | 6-phosphofructo-2-kinase | 1.29E-02 |
| DPOGS210627 | CG3940 | CG3940 | 1.33E-02 |
| DPOGS212492 | CG31324 | CG31324 | 1.33E-02 |
| DPOGS208868 | Membrin | Membrin | 1.38E-02 |
| DPOGS214481 | Wnk | Wnk kinase | 1.38E-02 |
| DPOGS212884 | CG44153 | CG44153 | 1.38E-02 |
| DPOGS207942 | CG10660 | CG10660 | 1.44E-02 |
| DPOGS213007 | Cyp18a1 | Cytochrome P450-18a1 | 1.44E-02 |
| DPOGS209874 | CG33281 | CG33281 | 1.60E-02 |
| DPOGS206692 | pdgy | pudgy | 1.61E-02 |
| DPOGS208120 | CG8545 | CG8545 | 1.61E-02 |
| DPOGS213327 | CG7110 | CG7110 | 1.76E-02 |
| DPOGS205823 | CG33791 | CG33791 | 1.94E-02 |
| DPOGS209925 | cwo | clockwork orange | 1.94E-02 |
| DPOGS215160 | Tret1-2 | Trehalose transporter 1-2 | 1.94E-02 |
| DPOGS209797 | Vha100-2 | Vacuolar H[+] ATPase 100kD subunit 2 | 1.98E-02 |
| DPOGS205264 | CG7470 | CG7470 | 1.98E-02 |
| DPOGS208406 | CG30069 | CG30069 | 1.99E-02 |
| DPOGS212590 | ninaB | neither inactivation nor afterpotential B | 2.04E-02 |
| DPOGS212327 | CG11658 | CG11658 | 2.11E-02 |
| DPOGS214408 | CG42269 | CG42269 | 2.11E-02 |
| DPOGS207444 | CG42237 | CG42237 | 2.11E-02 |
| DPOGS214070 | stumps | stumps | 2.25E-02 |
| DPOGS205927 | spen | split ends | 2.25E-02 |
| DPOGS212844 | Ctr1A | Copper transporter 1A | 2.28E-02 |
| DPOGS213804 | IP3K1 | Inositol 1,4,5-triphosphate kinase 1 | 2.33E-02 |
| DPOGS209591 | osp | outspread | 2.44E-02 |
| DPOGS200190 | sima | similar | 2.45E-02 |
| DPOGS214162 | Nha1 | Na[+]/H[+] hydrogen antiporter 1 | 2.45E-02 |
| DPOGS207764 | Eno | Enolase | 2.53E-02 |
| DPOGS215738 | egr | eiger | 2.53E-02 |
| DPOGS202827 | santa-maria | scavenger receptor acting in neural tissue and majority of rhodopsin is absent | 2.53E-02 |
| DPOGS201488 | CG2765 | CG2765 | 2.53E-02 |
| DPOGS205911 | Vps20 | Vacuolar protein sorting 20 | 2.53E-02 |
| DPOGS209025 | CG43427 | CG43427 | 2.68E-02 |
| DPOGS206893 | Snap29 | Synaptosomal-associated protein 29kDa | 2.68E-02 |
| DPOGS208596 | CG15186 | CG15186 | 2.75E-02 |
| DPOGS213502 | Fdx1 | Ferredoxin 1 | 2.79E-02 |
| DPOGS213207 | CG1667 | CG1667 | 2.80E-02 |
| DPOGS209166 | Bre1 | Bre1 | 2.81E-02 |
| DPOGS213913 | CG4797 | CG4797 | 2.81E-02 |
| DPOGS203868 | CG43693 | CG43693 | 2.86E-02 |
| DPOGS200089 | Tpi | Triose phosphate isomerase | 2.88E-02 |
| DPOGS214927 | Sin3A | Sin3A | 2.88E-02 |
| DPOGS200426 | CG30460 | CG30460 | 3.07E-02 |
| DPOGS205647 | CG17646 | CG17646 | 3.10E-02 |
| DPOGS205079 | CG7632 | CG7632 | 3.13E-02 |
| DPOGS207274 | Socs16D | Suppressor of Cytokine Signaling at 16D | 3.17E-02 |
| DPOGS205105 | CG4822 | CG4822 | 3.17E-02 |
| DPOGS203890 | santa-maria | scavenger receptor acting in neural tissue and majority of rhodopsin is absent | 3.17E-02 |
| DPOGS212605 | CG5853 | CG5853 | 3.19E-02 |
| DPOGS213576 | Hexo2 | Hexosaminidase 2 | 3.32E-02 |
| DPOGS207222 | robl | roadblock | 3.40E-02 |
| DPOGS200883 | CG16791 | CG16791 | 3.43E-02 |
| DPOGS206043 | ftz-f1 | ftz transcription factor 1 | 3.44E-02 |
| DPOGS207057 | CG45065 | CG45065 | 3.44E-02 |
| DPOGS215494 | AGBE | 1,4-Alpha-Glucan Branching Enzyme | 3.45E-02 |
| DPOGS212858 | atl | atlastin | 3.66E-02 |
| DPOGS209508 | Pgm | phosphoglucose mutase | 3.66E-02 |
| DPOGS205706 | CG42588 | CG42588 | 3.66E-02 |
| DPOGS208306 | Ance-3 | Ance-3 | 3.66E-02 |
| DPOGS209035 | CG8036 | CG8036 | 3.66E-02 |
| DPOGS214921 | Mco1 | Multicopper oxidase-1 | 3.67E-02 |
| DPOGS212022 | klar | klarsicht | 3.67E-02 |
| DPOGS202237 | CG5001 | CG5001 | 3.69E-02 |
| DPOGS204642 | Graf | GTPase regulator associated with FAK | 3.71E-02 |
| DPOGS200811 | h | hairy | 3.80E-02 |
| DPOGS207448 | Best1 | Bestrophin 1 | 3.86E-02 |
| DPOGS215969 | Taldo | Transaldolase | 4.07E-02 |
| DPOGS202213 | CG6443 | CG6443 | 4.07E-02 |
| DPOGS211148 | uzip | unzipped | 4.09E-02 |
| DPOGS214215 | Nha1 | Na[+]/H[+] hydrogen antiporter 1 | 4.09E-02 |
| DPOGS213288 | Nab2 | Nuclear polyadenosine RNA-binding 2 | 4.28E-02 |
| DPOGS208188 | Sema5c | Semaphorin 5c | 4.38E-02 |
| DPOGS215159 | Tret1-1 | Trehalose transporter 1-1 | 4.38E-02 |
| DPOGS210492 | Cpsf73 | Cleavage and polyadenylation specificity factor 73 | 4.39E-02 |
| DPOGS203710 | CG8602 | CG8602 | 4.42E-02 |
| DPOGS203970 | DUBAI | Deubiquitinating apoptotic inhibitor | 4.48E-02 |
| DPOGS209175 | CG14945 | CG14945 | 4.57E-02 |
| DPOGS212595 | Tps1 | Trehalose-6-phosphate synthase 1 | 4.60E-02 |
| DPOGS202434 | e | ebony | 4.65E-02 |
| DPOGS214099 | CG3792 | CG3792 | 4.68E-02 |
| DPOGS205027 | GlyP | Glycogen phosphorylase | 4.69E-02 |
| DPOGS215460 | Gapdh2 | Glyceraldehyde 3 phosphate dehydrogenase 2 | 4.82E-02 |
| DPOGS207871 | Grip163 | Grip163 | 4.82E-02 |
| DPOGS202815 | Eaat2 | Excitatory amino acid transporter 2 | 4.91E-02 |
| DPOGS204181 | Fit1 | Fermitin 1 | 4.92E-02 |
