# Supplementary material for: Genome-wide discovery of the daily transcriptome, DNA regulatory elements and transcription factor occupancy in the monarch butterfly brain
Source: PLoS Genet. 2019 Jul 23;15(7):e1008265. doi: 10.1371/journal.pgen.1008265 (PMC6677324; doi:10.1371/journal.pgen.1008265)
Supplement: S4 Table — (DOCX) [file pgen.1008265.s004.docx]

**S4 Table.** Rhythmic genes in wild-type differentially expressed in *Clk* knockouts with adjusted *p*-value (adjP) ≤ 0.05 from robust DODR method.

| **geneID** | **symbol** | **name** | **adjP** |
| --- | --- | --- | --- |
| DPOGS207942 | CG10660 | CG10660 | 8.51E-05 |
| DPOGS213900 | Hsp68 | Heat shock protein 68 | 2.54E-04 |
| DPOGS207000 | Mhcl | Myosin heavy chain-like | 2.54E-04 |
| DPOGS210128 | Papss | PAPS synthetase | 2.54E-04 |
| DPOGS211121 | CG4502 | CG4502 | 6.28E-04 |
| DPOGS205557 | Cenp-C | Centromeric protein-C | 7.10E-04 |
| DPOGS211461 | CG8312 | CG8312 | 1.41E-03 |
| DPOGS204644 | CG11438 | CG11438 | 1.41E-03 |
| DPOGS204253 | CG32032 | CG32032 | 1.41E-03 |
| DPOGS213114 | Ctl2 | Choline transporter-like 2 | 1.41E-03 |
| DPOGS203908 | per | period | 1.41E-03 |
| DPOGS211148 | uzip | unzipped | 1.41E-03 |
| DPOGS215479 | CG5235 | CG5235 | 1.46E-03 |
| DPOGS209508 | Pgm | phosphoglucose mutase | 1.51E-03 |
| DPOGS201544 | Oatp74D | Organic anion transporting polypeptide 74D | 1.52E-03 |
| DPOGS213901 | Hsp68 | Heat shock protein 68 | 1.76E-03 |
| DPOGS202993 | CG7888 | CG7888 | 1.81E-03 |
| DPOGS207730 | CG10082 | CG10082 | 2.03E-03 |
| DPOGS215720 | Argk | Arginine kinase | 2.07E-03 |
| DPOGS213925 | Hsp68 | Heat shock protein 68 | 2.07E-03 |
| DPOGS205027 | GlyP | Glycogen phosphorylase | 2.21E-03 |
| DPOGS212829 | CG10175 | CG10175 | 2.28E-03 |
| DPOGS208406 | CG30069 | CG30069 | 2.48E-03 |
| DPOGS215969 | Taldo | Transaldolase | 2.48E-03 |
| DPOGS202602 | Mcm2 | Minichromosome maintenance 2 | 2.51E-03 |
| DPOGS211900 | aop | anterior open | 2.72E-03 |
| DPOGS200356 | HEATR2 | HEAT repeat containing 2 | 2.74E-03 |
| DPOGS215494 | AGBE | 1,4-Alpha-Glucan Branching Enzyme | 2.87E-03 |
| DPOGS201194 | CG5535 | CG5535 | 2.87E-03 |
| DPOGS206348 | Picot | Picot | 3.43E-03 |
| DPOGS214070 | stumps | stumps | 3.45E-03 |
| DPOGS207764 | Eno | Enolase | 3.46E-03 |
| DPOGS209874 | CG33281 | CG33281 | 3.54E-03 |
| DPOGS205549 | CG43795 | CG43795 | 3.54E-03 |
| DPOGS212844 | Ctr1A | Copper transporter 1A | 3.76E-03 |
| DPOGS208881 | Droj2 | DnaJ-like-2 | 3.86E-03 |
| DPOGS210295 | Pgi | Phosphoglucose isomerase | 3.86E-03 |
| DPOGS209035 | CG8036 | CG8036 | 4.01E-03 |
| DPOGS202126 | CG32432 | CG32432 | 4.01E-03 |
| DPOGS202781 | Hop | Hsp70/Hsp90 organizing protein | 4.01E-03 |
| DPOGS208606 | vri | vrille | 4.01E-03 |
| DPOGS207058 | CG9518 | CG9518 | 4.08E-03 |
| DPOGS205823 | CG33791 | CG33791 | 4.17E-03 |
| DPOGS215738 | egr | eiger | 4.17E-03 |
| DPOGS205833 | CG9485 | CG9485 | 4.49E-03 |
| DPOGS204673 | CG13813 | CG13813 | 4.49E-03 |
| DPOGS207651 | CG17323 | CG17323 | 4.49E-03 |
| DPOGS211388 | CG2082 | CG2082 | 4.83E-03 |
| DPOGS209166 | Bre1 | Bre1 | 4.83E-03 |
| DPOGS212608 | CG11601 | CG11601 | 4.83E-03 |
| DPOGS209175 | CG14945 | CG14945 | 4.83E-03 |
| DPOGS204626 | CG7582 | CG7582 | 4.83E-03 |
| DPOGS200490 | Mdh1 | Malate dehydrogenase 1 | 5.38E-03 |
| DPOGS215384 | RIOK2 | RIO kinase 2 | 5.38E-03 |
| DPOGS202178 | Got1 | Glutamate oxaloacetate transaminase 1 | 5.70E-03 |
| DPOGS204494 | GABA-B-R1 | metabotropic GABA-B receptor subtype 1 | 5.87E-03 |
| DPOGS201345 | CG5958 | CG5958 | 6.09E-03 |
| DPOGS206596 | mgl | Megalin | 6.42E-03 |
| DPOGS212976 | Pdh | Photoreceptor dehydrogenase | 6.52E-03 |
| DPOGS215460 | Gapdh2 | Glyceraldehyde 3 phosphate dehydrogenase 2 | 7.06E-03 |
| DPOGS214042 | beta-Spec | beta Spectrin | 7.81E-03 |
| DPOGS208217 | Unc-115a | Uncoordinated 115a | 7.81E-03 |
| DPOGS206646 | Sik2 | Salt-inducible kinase 2 | 8.18E-03 |
| DPOGS200089 | Tpi | Triose phosphate isomerase | 8.18E-03 |
| DPOGS209025 | CG43427 | CG43427 | 9.51E-03 |
| DPOGS210257 | e | ebony | 9.51E-03 |
| DPOGS212996 | ImpE1 | Ecdysone-inducible gene E1 | 9.51E-03 |
| DPOGS203797 | Hsf | Heat shock factor | 9.51E-03 |
| DPOGS201376 | Neurl4 | Neuralized E3 ubiquitin protein ligase 4 | 9.51E-03 |
| DPOGS203808 | a | arc | 1.05E-02 |
| DPOGS201241 | CAP | CAP | 1.05E-02 |
| DPOGS212595 | Tps1 | Trehalose-6-phosphate synthase 1 | 1.05E-02 |
| DPOGS200764 | wun | wunen | 1.05E-02 |
| DPOGS213546 | CG10089 | CG10089 | 1.06E-02 |
| DPOGS209834 | CG14516 | CG14516 | 1.15E-02 |
| DPOGS202607 | mamo | maternal gene required for meiosis | 1.19E-02 |
| DPOGS203810 | Pfk | Phosphofructokinase | 1.21E-02 |
| DPOGS210186 | Pdk | Pyruvate dehydrogenase kinase | 1.21E-02 |
| DPOGS200426 | CG30460 | CG30460 | 1.27E-02 |
| DPOGS214402 | GlyS | Glycogen synthase | 1.27E-02 |
| DPOGS214179 | tim | timeless | 1.28E-02 |
| DPOGS213064 | Pgk | Phosphoglycerate kinase | 1.30E-02 |
| DPOGS208868 | Membrin | Membrin | 1.32E-02 |
| DPOGS208405 | CG30069 | CG30069 | 1.32E-02 |
| DPOGS208609 | Adf1 | Adh transcription factor 1 | 1.33E-02 |
| DPOGS212327 | CG11658 | CG11658 | 1.46E-02 |
| DPOGS213594 | Gbs-76A | Glycogen binding subunit 76A | 1.46E-02 |
| DPOGS201813 | Pglym78 | Phosphoglyceromutase | 1.46E-02 |
| DPOGS201445 | CG14984 | CG14984 | 1.56E-02 |
| DPOGS205079 | CG7632 | CG7632 | 1.56E-02 |
| DPOGS209925 | cwo | clockwork orange | 1.56E-02 |
| DPOGS203775 | NfI | Nuclear factor I | 1.56E-02 |
| DPOGS209167 | Ssadh | Succinic semialdehyde dehydrogenase | 1.69E-02 |
| DPOGS201195 | CG5535 | CG5535 | 1.72E-02 |
| DPOGS201012 | Lrpprc2 | Leucine-rich pentatricopeptide repeat containing 2 | 1.72E-02 |
| DPOGS210627 | CG3940 | CG3940 | 1.77E-02 |
| DPOGS208883 | Gpdh | Glycerol 3 phosphate dehydrogenase | 1.77E-02 |
| DPOGS216202 | cas | castor | 1.80E-02 |
| DPOGS204547 | Liprin-gamma | Liprin-gamma | 1.91E-02 |
| DPOGS215377 | CG9220 | CG9220 | 1.98E-02 |
| DPOGS214349 | wun | wunen | 1.98E-02 |
| DPOGS214921 | Mco1 | Multicopper oxidase-1 | 1.98E-02 |
| DPOGS202245 | Sras | severas | 1.98E-02 |
| DPOGS214481 | Wnk | Wnk kinase | 1.98E-02 |
| DPOGS212022 | klar | klarsicht | 1.98E-02 |
| DPOGS204181 | Fit1 | Fermitin 1 | 2.02E-02 |
| DPOGS204024 | Mocs2 | Molybdenum cofactor synthesis 2 | 2.02E-02 |
| DPOGS213007 | Cyp18a1 | Cytochrome P450-18a1 | 2.03E-02 |
| DPOGS214041 | beta-Spec | beta Spectrin | 2.05E-02 |
| DPOGS202608 | mamo | maternal gene required for meiosis | 2.17E-02 |
| DPOGS208185 | Scsalpha | Succinyl coenzyme A synthetase alpha subunit | 2.20E-02 |
| DPOGS209585 | CG8176 | CG8176 | 2.30E-02 |
| DPOGS208999 | CG7675 | CG7675 | 2.31E-02 |
| DPOGS215784 | nw | narrow | 2.60E-02 |
| DPOGS214482 | NA | No annotation | 2.60E-02 |
| DPOGS206136 | CG32369 | CG32369 | 2.63E-02 |
| DPOGS214408 | CG42269 | CG42269 | 2.63E-02 |
| DPOGS205824 | pigs | pickled eggs | 2.63E-02 |
| DPOGS205291 | sxc | super sex combs | 2.63E-02 |
| DPOGS215489 | Pfrx | 6-phosphofructo-2-kinase | 2.68E-02 |
| DPOGS213552 | Eip71CD | Ecdysone-induced protein 28/29kD | 2.69E-02 |
| DPOGS205927 | spen | split ends | 2.74E-02 |
| DPOGS202237 | CG5001 | CG5001 | 2.75E-02 |
| DPOGS213057 | Drip | Drip | 2.75E-02 |
| DPOGS212590 | ninaB | neither inactivation nor afterpotential B | 2.75E-02 |
| DPOGS207077 | CG13366 | CG13366 | 2.80E-02 |
| DPOGS205265 | CG13315 | CG13315 | 2.95E-02 |
| DPOGS201446 | CG10527 | CG10527 | 3.05E-02 |
| DPOGS200691 | E(spl)mbeta-HLH | Enhancer of split mbeta, helix-loop-helix | 3.09E-02 |
| DPOGS201894 | Mal-A4 | Maltase A4 | 3.15E-02 |
| DPOGS200407 | muc | midline uncoordinated | 3.21E-02 |
| DPOGS201013 | CG13868 | CG13868 | 3.33E-02 |
| DPOGS206382 | l(2)efl | lethal (2) essential for life | 3.48E-02 |
| DPOGS211191 | Zip71B | Zinc/iron regulated transporter-related protein 71B | 3.48E-02 |
| DPOGS206959 | Ald | Aldolase | 3.52E-02 |
| DPOGS203088 | CG7720 | CG7720 | 3.52E-02 |
| DPOGS215439 | l(2)efl | lethal (2) essential for life | 3.52E-02 |
| DPOGS213560 | nuf | nuclear fallout | 3.52E-02 |
| DPOGS202827 | santa-maria | scavenger receptor acting in neural tissue and majority of rhodopsin is absent | 3.52E-02 |
| DPOGS212889 | CG1218 | CG1218 | 3.57E-02 |
| DPOGS202434 | e | ebony | 3.57E-02 |
| DPOGS202815 | Eaat2 | Excitatory amino acid transporter 2 | 3.57E-02 |
| DPOGS210302 | Myb | Myb oncogene-like | 3.57E-02 |
| DPOGS209948 | regucalcin | regucalcin | 3.57E-02 |
| DPOGS207274 | Socs16D | Suppressor of Cytokine Signaling at 16D | 3.57E-02 |
| DPOGS213632 | pall | pallbearer | 3.61E-02 |
| DPOGS203996 | RhoGAP15B | Rho GTPase activating protein at 15B | 3.61E-02 |
| DPOGS209143 | CG7458 | CG7458 | 3.65E-02 |
| DPOGS200817 | CG5150 | CG5150 | 3.70E-02 |
| DPOGS200985 | Lgr1 | Leucine-rich repeat-containing G protein-coupled receptor 1 | 3.73E-02 |
| DPOGS201162 | Esp | Epidermal stripes and patches | 3.75E-02 |
| DPOGS215488 | CG7376 | CG7376 | 3.92E-02 |
| DPOGS201651 | NC2alpha | Negative Cofactor 2alpha | 3.97E-02 |
| DPOGS213327 | CG7110 | CG7110 | 4.16E-02 |
| DPOGS215160 | Tret1-2 | Trehalose transporter 1-2 | 4.33E-02 |
| DPOGS206473 | CG4797 | CG4797 | 4.56E-02 |
| DPOGS200883 | CG16791 | CG16791 | 4.56E-02 |
| DPOGS201488 | CG2765 | CG2765 | 4.56E-02 |
| DPOGS204552 | CG1213 | CG1213 | 4.82E-02 |
| DPOGS214879 | pn | prune | 4.82E-02 |
| DPOGS201429 | cac | cacophony | 4.88E-02 |
| DPOGS202114 | crol | crooked legs | 4.88E-02 |
| DPOGS209507 | SamDC | S-adenosylmethionine decarboxylase | 4.90E-02 |
