# Supplementary material for: Genome-wide discovery of the daily transcriptome, DNA regulatory elements and transcription factor occupancy in the monarch butterfly brain
Source: PLoS Genet. 2019 Jul 23;15(7):e1008265. doi: 10.1371/journal.pgen.1008265 (PMC6677324; doi:10.1371/journal.pgen.1008265)
Supplement: S5 Table — (DOCX) [file pgen.1008265.s005.docx]

**S5 Table.** Rhythmic genes in wild-type that are differentially expressed both in *Cry2* knockouts and *Clk* knockouts with adjusted *p*-value (adjP1 for wild-type vs. *Cry2* knockouts, and adjP2 for wild-type vs. *Clk* knockouts) ≤ 0.05 from robust DODR method.

| **gene ID** | **gene symbol** | **gene name** | **adjP 1** | **adjP 2** |
| --- | --- | --- | --- | --- |
| DPOGS200089 | Tpi | Triose phosphate isomerase | 0.0288 | 0.0082 |
| DPOGS200426 | CG30460 | CG30460 | 0.0307 | 0.0127 |
| DPOGS200490 | Mdh1 | Malate dehydrogenase 1 | 0.0037 | 0.0054 |
| DPOGS200691 | E(spl)mbeta-HLH | Enhancer of split mbeta, helix-loop-helix | 0.0080 | 0.0309 |
| DPOGS200883 | CG16791 | CG16791 | 0.0343 | 0.0456 |
| DPOGS201012 | Lrpprc2 | Leucine-rich pentatricopeptide repeat containing 2 | 0.0046 | 0.0172 |
| DPOGS201013 | CG13868 | CG13868 | 0.0109 | 0.0333 |
| DPOGS201195 | CG5535 | CG5535 | 0.0059 | 0.0172 |
| DPOGS201488 | CG2765 | CG2765 | 0.0253 | 0.0456 |
| DPOGS201544 | Oatp74D | Organic anion transporting polypeptide 74D | 0.0036 | 0.0015 |
| DPOGS202237 | CG5001 | CG5001 | 0.0369 | 0.0275 |
| DPOGS202434 | e | ebony | 0.0465 | 0.0357 |
| DPOGS202815 | Eaat2 | Excitatory amino acid transporter 2 | 0.0491 | 0.0357 |
| DPOGS202827 | santa-maria | scavenger receptor acting in neural tissue and majority of rhodopsin is absent | 0.0253 | 0.0352 |
| DPOGS203088 | CG7720 | CG7720 | 0.0114 | 0.0352 |
| DPOGS203797 | Hsf | Heat shock factor | 0.0029 | 0.0095 |
| DPOGS203810 | Pfk | Phosphofructokinase | 0.0087 | 0.0121 |
| DPOGS203908 | per | period | 0.0006 | 0.0014 |
| DPOGS204181 | Fit1 | Fermitin 1 | 0.0492 | 0.0202 |
| DPOGS204253 | CG32032 | CG32032 | 0.0020 | 0.0014 |
| DPOGS204644 | CG11438 | CG11438 | 0.0010 | 0.0014 |
| DPOGS205027 | GlyP | Glycogen phosphorylase | 0.0469 | 0.0022 |
| DPOGS205079 | CG7632 | CG7632 | 0.0313 | 0.0156 |
| DPOGS205549 | CG43795 | CG43795 | 0.0021 | 0.0035 |
| DPOGS205823 | CG33791 | CG33791 | 0.0194 | 0.0042 |
| DPOGS205927 | spen | split ends | 0.0225 | 0.0274 |
| DPOGS207000 | Mhcl | Myosin heavy chain-like | 0.0006 | 0.0003 |
| DPOGS207058 | CG9518 | CG9518 | 0.0014 | 0.0041 |
| DPOGS207274 | Socs16D | Suppressor of Cytokine Signaling at 16D | 0.0317 | 0.0357 |
| DPOGS207730 | CG10082 | CG10082 | 0.0029 | 0.0020 |
| DPOGS207764 | Eno | Enolase | 0.0253 | 0.0035 |
| DPOGS207942 | CG10660 | CG10660 | 0.0144 | 0.0001 |
| DPOGS208406 | CG30069 | CG30069 | 0.0199 | 0.0025 |
| DPOGS208606 | vri | vrille | 0.0050 | 0.0040 |
| DPOGS208868 | Membrin | Membrin | 0.0138 | 0.0132 |
| DPOGS209025 | CG43427 | CG43427 | 0.0268 | 0.0095 |
| DPOGS209035 | CG8036 | CG8036 | 0.0366 | 0.0040 |
| DPOGS209166 | Bre1 | Bre1 | 0.0281 | 0.0048 |
| DPOGS209175 | CG14945 | CG14945 | 0.0457 | 0.0048 |
| DPOGS209508 | Pgm | phosphoglucose mutase | 0.0366 | 0.0015 |
| DPOGS209874 | CG33281 | CG33281 | 0.0160 | 0.0035 |
| DPOGS209925 | cwo | clockwork orange | 0.0194 | 0.0156 |
| DPOGS210128 | Papss | PAPS synthetase | 0.0010 | 0.0003 |
| DPOGS210257 | e | ebony | 0.0033 | 0.0095 |
| DPOGS210627 | CG3940 | CG3940 | 0.0133 | 0.0177 |
| DPOGS211121 | CG4502 | CG4502 | 0.0016 | 0.0006 |
| DPOGS211148 | uzip | unzipped | 0.0409 | 0.0014 |
| DPOGS212022 | klar | klarsicht | 0.0367 | 0.0198 |
| DPOGS212327 | CG11658 | CG11658 | 0.0211 | 0.0146 |
| DPOGS212590 | ninaB | neither inactivation nor afterpotential B | 0.0204 | 0.0275 |
| DPOGS212595 | Tps1 | Trehalose-6-phosphate synthase 1 | 0.0460 | 0.0105 |
| DPOGS212608 | CG11601 | CG11601 | 0.0083 | 0.0048 |
| DPOGS212844 | Ctr1A | Copper transporter 1A | 0.0228 | 0.0038 |
| DPOGS213007 | Cyp18a1 | Cytochrome P450-18a1 | 0.0144 | 0.0203 |
| DPOGS213064 | Pgk | Phosphoglycerate kinase | 0.0062 | 0.0130 |
| DPOGS213114 | Ctl2 | Choline transporter-like 2 | 0.0112 | 0.0014 |
| DPOGS213327 | CG7110 | CG7110 | 0.0176 | 0.0416 |
| DPOGS213552 | Eip71CD | Ecdysone-induced protein 28/29kD | 0.0019 | 0.0269 |
| DPOGS213900 | Hsp68 | Heat shock protein 68 | 0.0010 | 0.0003 |
| DPOGS213901 | Hsp68 | Heat shock protein 68 | 0.0020 | 0.0018 |
| DPOGS213925 | Hsp68 | Heat shock protein 68 | 0.0029 | 0.0021 |
| DPOGS214070 | stumps | stumps | 0.0225 | 0.0034 |
| DPOGS214179 | tim | timeless | 0.0010 | 0.0128 |
| DPOGS214408 | CG42269 | CG42269 | 0.0211 | 0.0263 |
| DPOGS214481 | Wnk | Wnk kinase | 0.0138 | 0.0198 |
| DPOGS214921 | Mco1 | Multicopper oxidase-1 | 0.0367 | 0.0198 |
| DPOGS215160 | Tret1-2 | Trehalose transporter 1-2 | 0.0194 | 0.0433 |
| DPOGS215460 | Gapdh2 | Glyceraldehyde 3 phosphate dehydrogenase 2 | 0.0482 | 0.0071 |
| DPOGS215489 | Pfrx | 6-phosphofructo-2-kinase | 0.0129 | 0.0268 |
| DPOGS215494 | AGBE | 1,4-Alpha-Glucan Branching Enzyme | 0.0345 | 0.0029 |
| DPOGS215738 | egr | eiger | 0.0253 | 0.0042 |
| DPOGS215969 | Taldo | Transaldolase | 0.0407 | 0.0025 |
