# Supplementary material for: Genome-wide discovery of the daily transcriptome, DNA regulatory elements and transcription factor occupancy in the monarch butterfly brain
Source: PLoS Genet. 2019 Jul 23;15(7):e1008265. doi: 10.1371/journal.pgen.1008265 (PMC6677324; doi:10.1371/journal.pgen.1008265)
Supplement: S6 Table — (DOCX) [file pgen.1008265.s006.docx]

**S6 Table.** Enriched Gene Ontology (GO) terms of biological processes for rhythmic genes in wild-type differentially expressed in (A) *Cry2* knockouts and (B) *Clk* knockouts.

**A**

| **Biological processes** | **GO identifiers** | **p-value** | **-Log10 (p-value)** |
| --- | --- | --- | --- |
| Pyridine nucleotide metabolic process | GO:0019362 | 1.9377 x 10^-8^ | 7.713 |
| Transmembrane transport | GO:0055085 | 1.5151 x 10^-5^ | 4.820 |
| Carbohydrate biosynthetic process | GO:0016051 | 7.1458 x 10^-4^ | 3.146 |
| Ecdysteroid metabolic process | GO:0045455 | 8.0282 x 10^-4^ | 3.0954 |
| Oxidation-reduction process | GO:0055114 | 1.556 x 10^-3^ | 2.808 |
| Multicellular organism aging | GO:0010259 | 1.908 x 10^-3^ | 2.720 |
| Regulation of DNA binding | GO:0051101 | 2.376 x 10^-3^ | 2.624 |
| Positive regulation of small molecule metabolic process | GO:0062013 | 2.850 x 10^-3^ | 2.545 |
| Cellular aldehyde metabolic process | GO:0006081 | 3.960 x 10^-3^ | 2.402 |
| Ion transport | GO:0006811 | 4.367 x 10^-3^ | 2.360 |

**B**

| **Biological processes** | **GO identifiers** | **p-value** | **-Log10 (p-value)** |
| --- | --- | --- | --- |
| Glycolytic process | GO:0006096 | 6.297 x 10^-13^ | 12.201 |
| Glycogen biosynthetic process | GO:0005978 | 1.476 x 10^-5^ | 4.831 |
| Oxidation-reduction process | GO:0055114 | 1.055 x 10^-4^ | 3.977 |
| Cellular aldehyde metabolic process | GO:0006081 | 6.763 x 10^-4^ | 3.170 |
| Regulation of heart contraction | GO:0008016 | 1.443 x 10^-3^ | 2.841 |
| Transmembrane transport | GO:0055085 | 2.997 x 10^-3^ | 2.523 |
| Regulation of DNA binding | GO:0051101 | 4.748 x 10^-3^ | 2.323 |
| Cellular biogenic amine metabolic process | GO:0006576 | 7.829 x 10^-3^ | 2.106 |
| Multicellular organism aging | GO:0010259 | 8.446 x 10^-3^ | 2.073 |
| Developmental pigmentation | GO:0048066 | 9.667 x 10^-3^ | 2.015 |
