# Supplementary material for: Genome-wide discovery of the daily transcriptome, DNA regulatory elements and transcription factor occupancy in the monarch butterfly brain
Source: PLoS Genet. 2019 Jul 23;15(7):e1008265. doi: 10.1371/journal.pgen.1008265 (PMC6677324; doi:10.1371/journal.pgen.1008265)
Supplement: S7 Table — (DOCX) [file pgen.1008265.s007.docx]

**S7 Table.** Enriched KEGG pathways for rhythmic genes in wild-type differentially expressed in (A) *Cry2* knockouts and (B) *Clk* knockouts.

**A**

| **KEGG pathways** | **KEGG identifiers** | **p-value** | **-Log10 (p-value)** |
| --- | --- | --- | --- |
| Biosynthesis of amino acids | dme01230 | 4.422 x 10^-7^ | 6.354 |
| Starch and sucrose metabolism | dme00500 | 1.302 x 10^-4^ | 3.885 |
| Pentose phosphate pathway | dme00030 | 2.132 x 10^-4^ | 3.671 |
| Circadian rhythm - fly | dme04711 | 2.628 x 10^-4^ | 3.580 |
| Fructose and mannose metabolism | dme00051 | 3.377 x 10^-3^ | 2.471 |

**B**

| **KEGG pathways** | **KEGG identifiers** | **p-value** | **-Log10 (p-value)** |
| --- | --- | --- | --- |
| Glycolysis (Embden-Meyerhof pathway), glucose => pyruvate | M00001 | 7.114 x 10^-12^ | 11.148 |
| Starch and sucrose metabolism | dme00500 | 5.054 x 10^-10^ | 9.296 |
| Pentose phosphate pathway | dme00030 | 1.535 x 10^-6^ | 5.814 |
| Circadian rhythm - fly | dme04711 | 5.404 x 10^-4^ | 3.267 |
| Fructose and mannose metabolism | dme00051 | 5.431 x 10^-4^ | 3.265 |
| Citrate cycle (TCA cycle) | dme00020 | 3.063 x 10^-3^ | 2.514 |
| Galactose metabolism | dme00052 | 5.674 x 10^-3^ | 2.246 |
