# Supplementary material for: Genome-wide discovery of the daily transcriptome, DNA regulatory elements and transcription factor occupancy in the monarch butterfly brain
Source: PLoS Genet. 2019 Jul 23;15(7):e1008265. doi: 10.1371/journal.pgen.1008265 (PMC6677324; doi:10.1371/journal.pgen.1008265)
Supplement: S8 Table — R: biological replicate. (DOCX) [file pgen.1008265.s008.docx]

**S8 Table.** Number of ATAC-seq peaks identified for individual and merged replicates in wild-type, *Clk* knockouts (KO) and *Cyc-like* mutants (mut) at ZT04 and at ZT16. R: biological replicate.

| **Individual replicate** | **Number of peaks** | **Merged replicates** | **Number of peaks** |
| --- | --- | --- | --- |
| WT ZT04 R1 | 23,357 | WT ZT04 R1 + R2 | 23,222 |
| WT ZT04 R2 | 25,683 |  |  |
| WT ZT16 R1 | 14,042 | WT ZT16 R1 + R2 | 13,555 |
| WT ZT16 R2 | 14,069 |  |  |
| *Clk* KO ZT04 R1 | 12,628 | *Clk* KO ZT04 R1 + R2 | 14,970 |
| *Clk* KO ZT04 R2 | 18,948 |  |  |
| *Clk* KO ZT16 R1 | 20,720 | *Clk* KO ZT16 R1 + R2 | 19,708 |
| *Clk* KO ZT16 R2 | 19,563 |  |  |
| *Cyc*-like mut ZT04 R1 | 21,295 | *Cyc*-like ZT04 R1 + R2 | 17,503 |
| *Cyc*-like mut ZT04 R2 | 16,304 |  |  |
| *Cyc*-like mut ZT16 R1 | 19,241 | *Cyc*-like ZT16 R1 + R2 | 16,736 |
| *Cyc*-like mut ZT16 R2 | 17,231 |  |  |
