# Supplementary material for: Genome-wide discovery of the daily transcriptome, DNA regulatory elements and transcription factor occupancy in the monarch butterfly brain
Source: PLoS Genet. 2019 Jul 23;15(7):e1008265. doi: 10.1371/journal.pgen.1008265 (PMC6677324; doi:10.1371/journal.pgen.1008265)
Supplement: S9 Table — Higher Tn5 integration depicts a Log2 fold-change > 0.3785 (fold-change > 1.3) and FDR < 0.05. (DOCX) [file pgen.1008265.s009.docx]

**S9 Table.** Differential ATAC-seq peak analysis. Higher Tn5 integration depicts a Log2 fold-change > 0.3785 (fold-change > 1.3) and FDR < 0.05.

|  |  | Genome-wide peaks (n = 26,867) | | Peaks in rhythmic genes differentially regulated in *Clk* KO (n = 339) | |
| --- | --- | --- | --- | --- | --- |
| A | B | Higher Tn5 integration in A | Higher Tn5 integration in B | Higher Tn5 integration in A | Higher Tn5 integration in B |
| WT ZT04 | WT ZT16 | 0 | 76 (0.283%) | 0 | 0 |
| WT ZT04 | *Clk* KO ZT04 | 33 (0.123%) | 170 (0.633%) | 1 (0.295%) | 2 (0.590%) |
| WT ZT04 | *Clk* KO ZT16 | 5 (0.019%) | 24 (0.089% | 0 | 0 |
| WT ZT04 | *Cyc-like* mut ZT04 | 0 | 22 (0.082%) | 0 | 0 |
| WT ZT04 | *Cyc-like* mut ZT16 | 2 (0.007%) | 98 (0.365%) | 0 | 1 (0.295%) |
| WT ZT16 | *Clk* KO ZT04 | 10 (0.037%) | 12 (0.045%) | 0 | 0 |
| WT ZT16 | *Clk* KO ZT16 | 41 (0.153%) | 4 (0.015%) | 1 (0.295%) | 0 |
| WT ZT16 | *Cyc-like* mut ZT04 | 2 (0.007%) | 14 (0.052%) | 0 | 0 |
| WT ZT16 | *Cyc-like* mut ZT16 | 10 (0.037%) | 32 (0.119%) | 0 | 0 |
| *Clk* KO ZT04 | *Clk* KO ZT16 | 3 (0.011%) | 2 (0.007%) | 0 | 0 |
| *Clk* KO ZT04 | *Cyc-like* mut ZT04 | 2 (0.007%) | 11 (0.041%) | 0 | 0 |
| *Clk* KO ZT04 | *Cyc-like* mut ZT16 | 1 (0.004%) | 2 (0.007%) | 0 | 0 |
| *Clk* KO ZT16 | *Cyc-like* mut ZT04 | 2 (0.007%) | 15 (0.056%) | 0 | 0 |
| *Clk* KO ZT16 | *Cyc-like* mut ZT16 | 1 (0.004%) | 16 (0.060%) | 0 | 0 |
| *Cyc-like* mut  ZT04 | *Cyc-like* mut ZT16 | 8 (0.030%) | 3 (0.011%) | 0 | 0 |
