# Supplementary material for: Genome-wide discovery of the daily transcriptome, DNA regulatory elements and transcription factor occupancy in the monarch butterfly brain
Source: PLoS Genet. 2019 Jul 23;15(7):e1008265. doi: 10.1371/journal.pgen.1008265 (PMC6677324; doi:10.1371/journal.pgen.1008265)
Supplement: S10 Table — R: biological replicate. (DOCX) [file pgen.1008265.s010.docx]

**S10 Table.** RNA-seq summary for wild-type (WT), *Cry2* knockouts (KO), and *Clk* KO. R: biological replicate.

| **Samples** | **Number of reads** | **Mapped reads** | **Mapping percentage** |
| --- | --- | --- | --- |
| WT ZT01 R1 | 8,329,736 | 7,474,898 | 89.7 |
| WT ZT01 R2 | 11,307,582 | 9,768,700 | 86.4 |
| WT ZT04 R1 | 8,308,713 | 7,353,942 | 88.5 |
| WT ZT04 R2 | 9,752,315 | 8,477,008 | 86.9 |
| WT ZT07 R1 | 7,489,482 | 6,551,179 | 87.5 |
| WT ZT07 R2 | 9,802,883 | 8,436,946 | 86.1 |
| WT ZT10 R1 | 8,292,323 | 7,183,739 | 86.6 |
| WT ZT10 R2 | 10,067,093 | 8,634,538 | 85.8 |
| WT ZT13 R1 | 7,414,155 | 6,488,395 | 87.5 |
| WT ZT13 R2 | 9,848,313 | 8,840,377 | 89.8 |
| WT ZT16 R1 | 8,373,408 | 7,151,758 | 85.4 |
| WT ZT16 R2 | 10,074,990 | 9,041,720 | 89.7 |
| WT ZT19 R1 | 8,023,590 | 6,893,871 | 85.9 |
| WT ZT19 R2 | 8,636,414 | 7,766,293 | 89.9 |
| WT ZT22 R1 | 8,279,696 | 7,136,863 | 86.2 |
| WT ZT22 R2 | 10,286,399 | 9,071,636 | 88.2 |
| *Cry2* KO ZT04 R1 | 9,987,045 | 8,914,479 | 89.3 |
| *Cry2* KO ZT04 R2 | 10,973,268 | 9,893,830 | 90.2 |
| *Cry2* KO ZT10 R1 | 8,139,974 | 7,361,729 | 90.4 |
| *Cry2* KO ZT10 R2 | 9,258,043 | 8,366,768 | 90.4 |
| *Cry2* KO ZT16 R1 | 8,498,634 | 7,617,395 | 89.6 |
| *Cry2* KO ZT16 R2 | 9,715,064 | 8,791,979 | 90.5 |
| *Cry2* KO ZT22 R1 | 7,918,255 | 7,194,359 | 90.9 |
| *Cry2* KO ZT22 R2 | 10,190,665 | 9,215,811 | 90.4 |
| *Clk* KO ZT04 R1 | 12,243,585 | 10,970,905 | 89.6 |
| *Clk* KO ZT04 R2 | 9,703,748 | 8,617,806 | 88.8 |
| *Clk* KO ZT10 R1 | 9,592,535 | 8,559,520 | 89.2 |
| *Clk* KO ZT10 R2 | 9,584,100 | 8,440,417 | 88.1 |
| *Clk* KO ZT16 R1 | 9,681,187 | 8,617,074 | 89.0 |
| *Clk* KO ZT16 R2 | 8,380,941 | 7,248,552 | 86.5 |
| *Clk* KO ZT22 R1 | 10,793,900 | 9,387,064 | 87.0 |
| *Clk* KO ZT22 R2 | 9,233,897 | 8,153,111 | 88.3 |
| **Average** | **9,318,185** | **8,238,208** | **88.4** |
