# Supplementary figures and images for: Genome-wide discovery of the daily transcriptome, DNA regulatory elements and transcription factor occupancy in the monarch butterfly brain
Source: PLoS Genet. 2019 Jul 23;15(7):e1008265. doi: 10.1371/journal.pgen.1008265 (PMC6677324; doi:10.1371/journal.pgen.1008265)

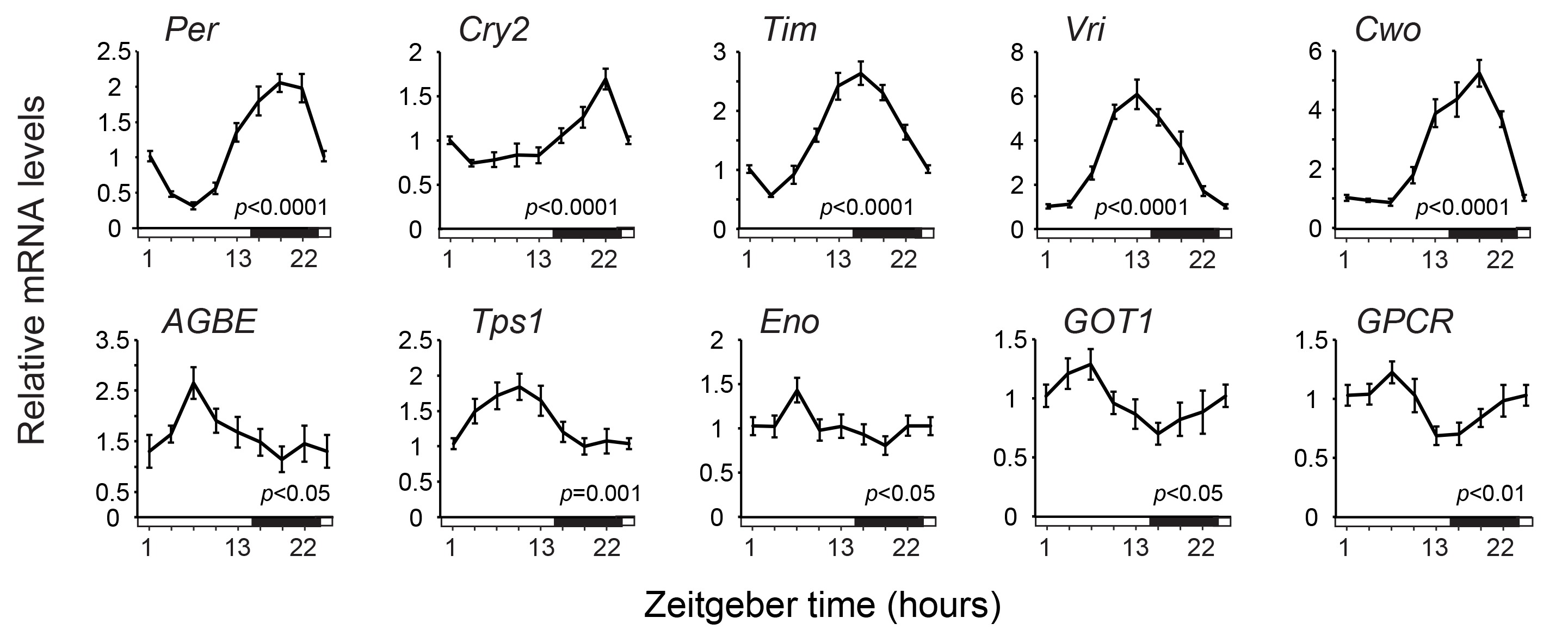

Supplement: S1 Fig — Diurnal expression of core clock genes (top) and a few other rhythmic candidate genes (bottom) in brains of wild-type monarchs entrained in 15:9 LD conditions. Values are mean ± SEM of six animals. Horizontal bars, day (white) and night (black). P-values, one-way ANOVA. Per, period; Cry2, cryptochrome 2; Tim, timeless; Vri, vrille; Cwo, clockworkorange; AGBE, 1,4-alpha-glucan branching enzyme; Tps1, trehalose-6-phosphate synthase; Eno, enolase; GOT1, glutamate oxaloacetate transaminase; GPCR, G-protein coupled receptor of unknown function. (TIF) [file pgen.1008265.s011.tif]

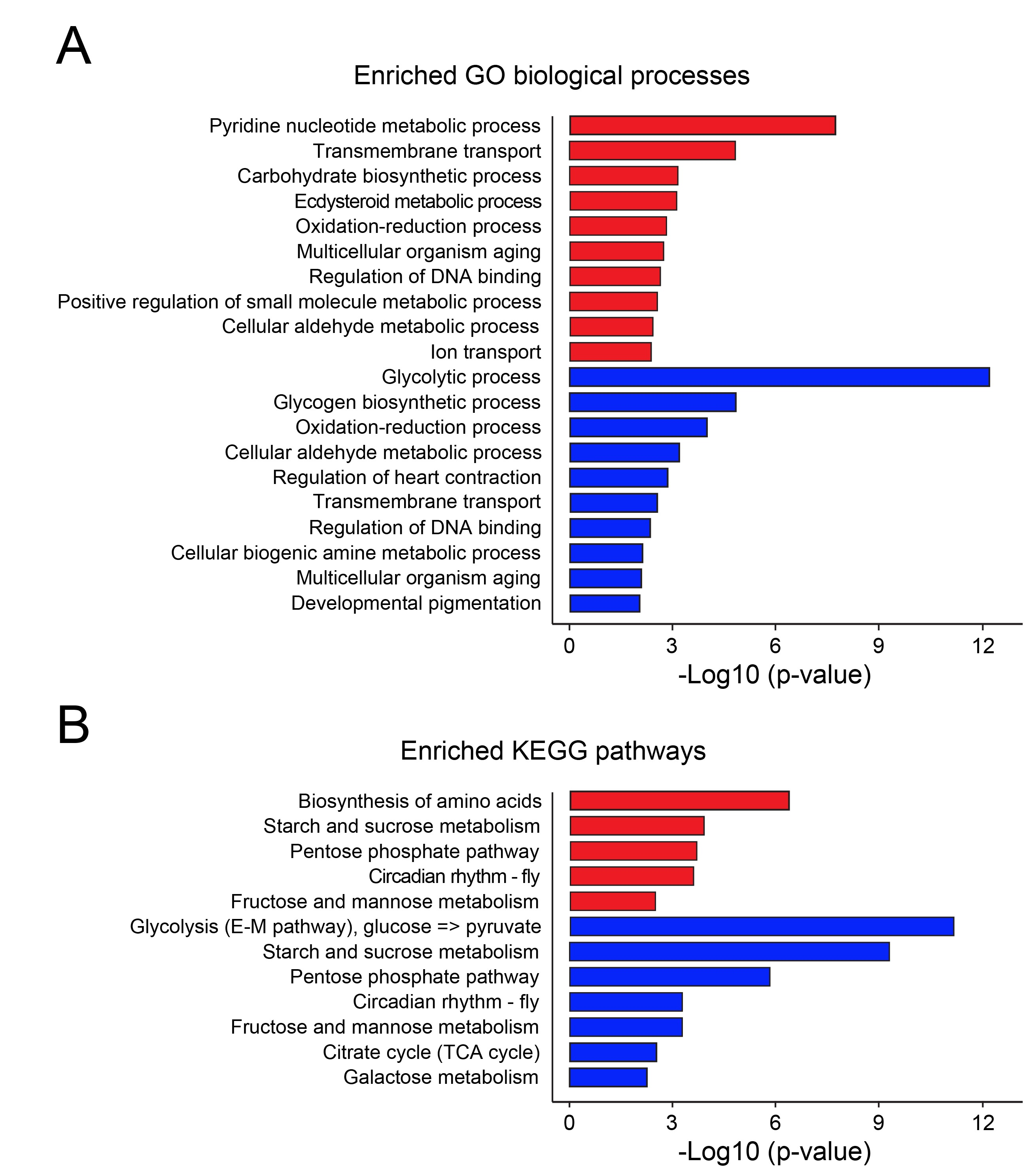

Supplement: S2 Fig — GO and KEGG pathways for genes with rhythmic expression levels in wild-type but differentially expressed in Cry2 and Clk knockouts, identified by Metascape at p <0.01, are shown in red and blue, respectively. (TIF) [file pgen.1008265.s012.tif]

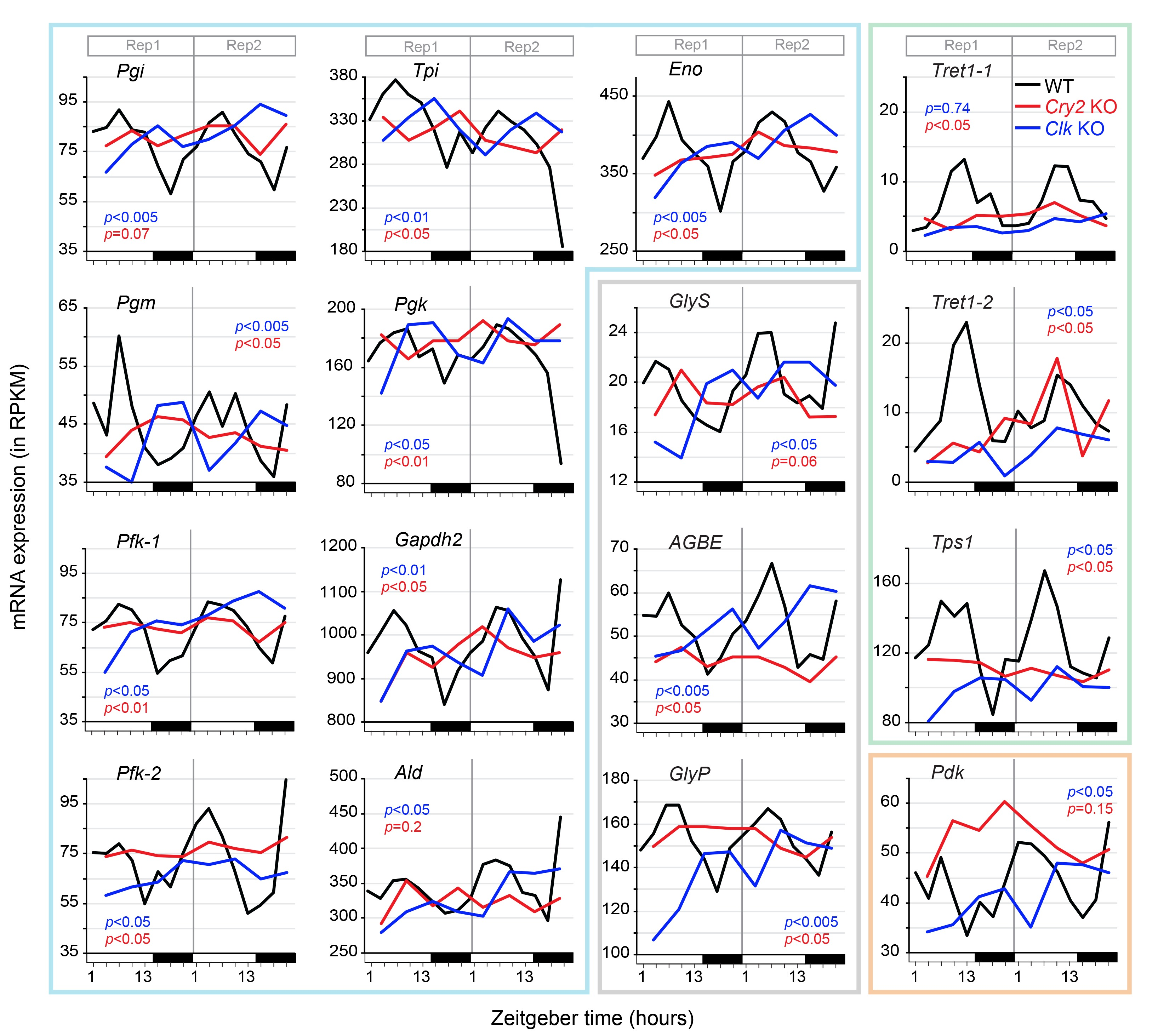

Supplement: S3 Fig — For each gene and each genotype, two biological replicates are plotted consecutively. Black line: wild-type; red line: Cry2 knockouts; blue line: Clk knockouts. mRNA expression levels are expressed in reads per kilobase of transcript per million reads mapped (RPKM). Tret1, trehalose transporter (Tret1-1 and Tret1-2); Tps1, trehalose-6-phosphate synthase; Pgi, phosphoglucose isomerase; Pfk-2/FBPase-2, 6-phosphofructo-2-kinase/fructose 2,6 biphosphatase; Pfk-1, 6-phosphofructokinase; Ald, aldolase; Tpi, triose phosphate isomerase; Gapdh2, glyceraldehyde 3 phosphate dehydrogenase 2; Pgk, phosphoglycerate kinase; Eno, enolase; Pdk, pyruvate dehydrogenase kinase; GlyS, glycogen synthase; AGBE: 1,4-alpha-glucan branching enzyme; GlyP, glycogen phosphorylase; Pgm, phosphoglucose mutase. p-values were obtained from p-values of the robust DODR [32] and corrected for multiple testing using the Benjamini-Hochberg method. (TIF) [file pgen.1008265.s013.tif]

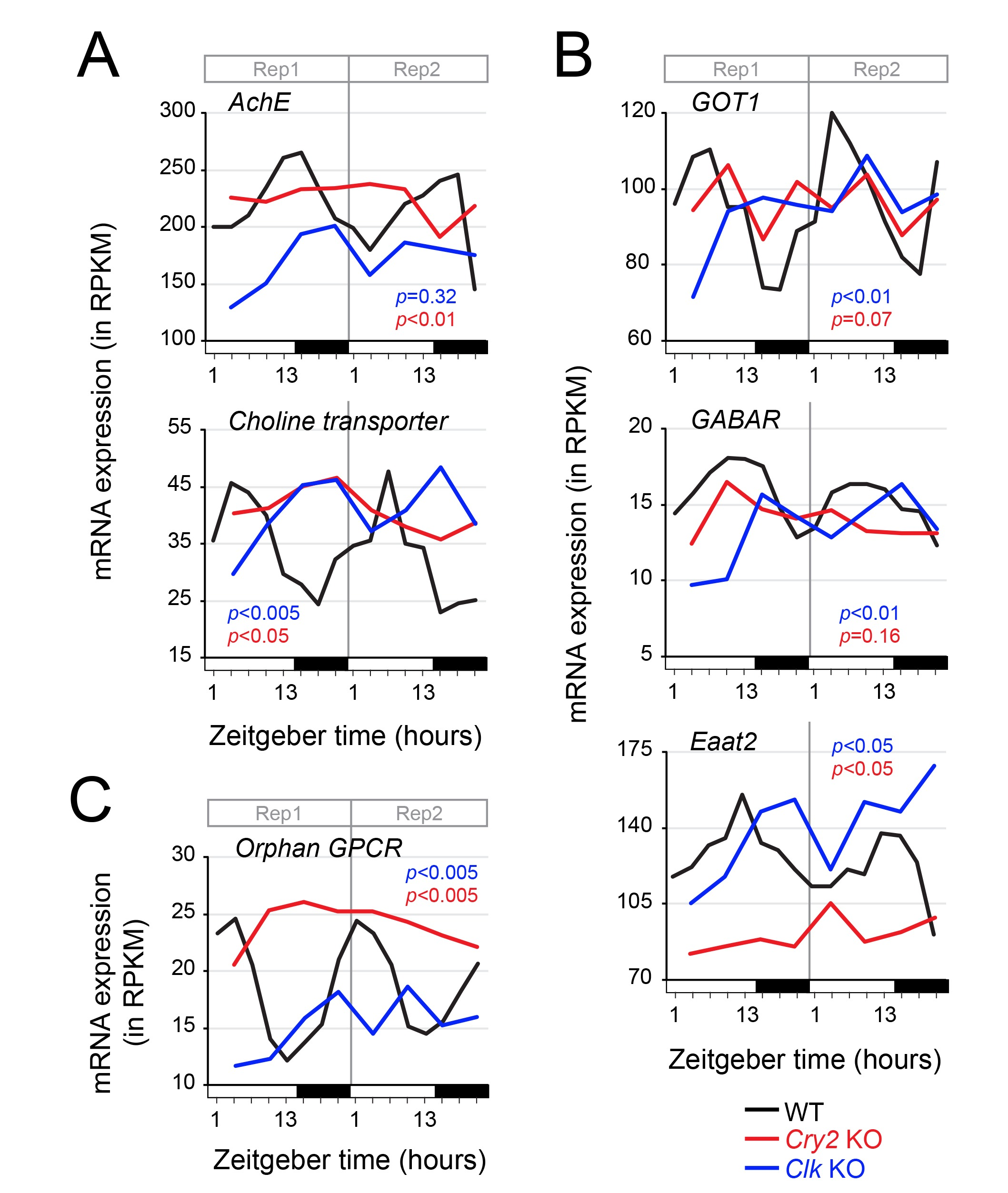

Supplement: S4 Fig — For each gene and each genotype, two biological replicates are plotted consecutively. Black line: wild-type; red line: Cry2 knockouts; blue line: Clk knockouts. mRNA expression levels are expressed in reads per kilobase of transcript per million reads mapped (RPKM). p-values were obtained from p-values of the robust DODR [32] and corrected for multiple testing using the Benjamini-Hochberg method. (TIF) [file pgen.1008265.s014.tif]

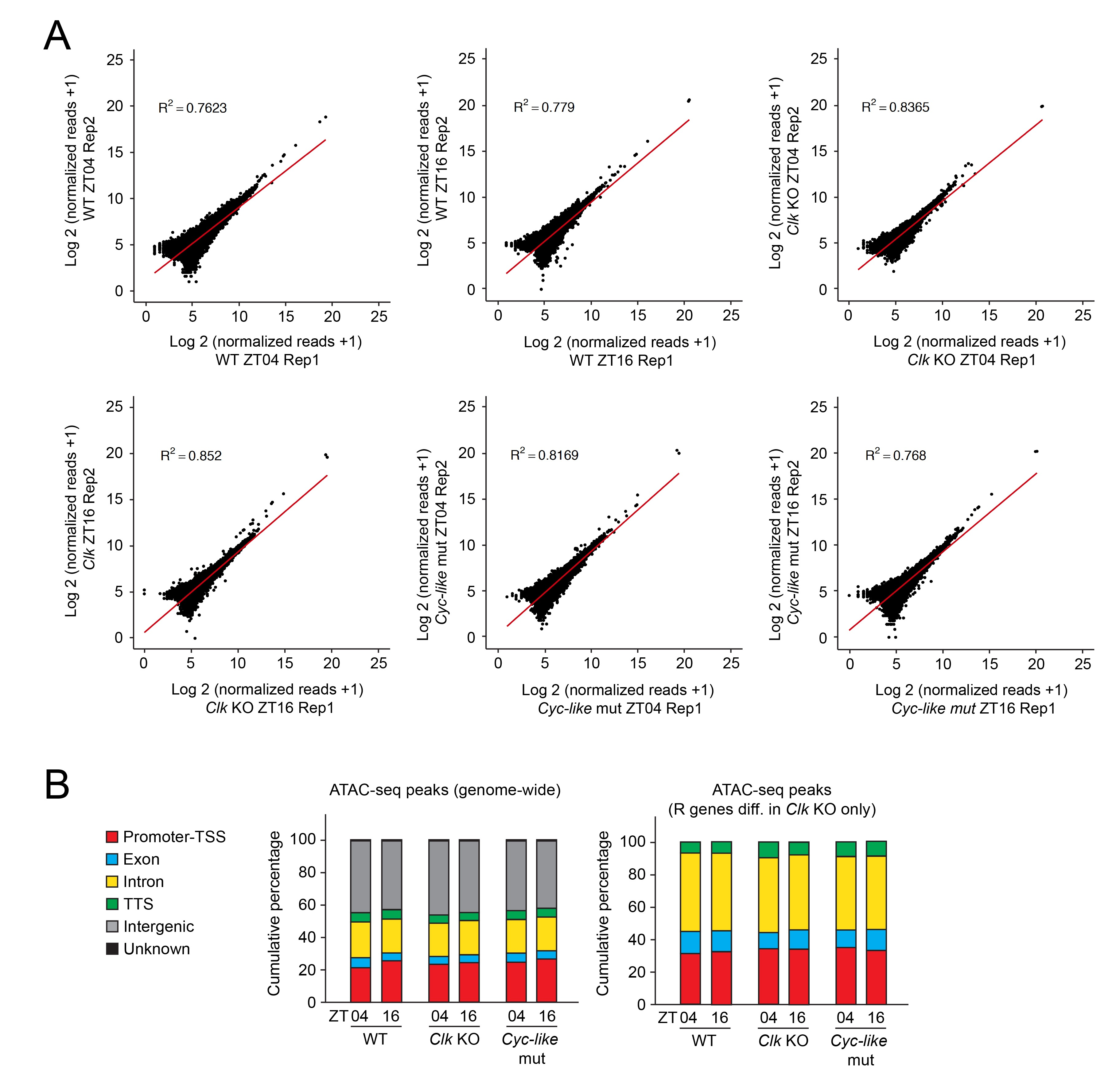

Supplement: S5 Fig — A) Scatter plots showing the ATAC-seq signal correlation between biological replicates at ZT04 and at ZT16 in brains of wild-type, Clk knockout and Cyc-like mutant monarchs. B) Distribution of ATAC-seq peaks in the monarch genome (left) and within -1Kb of the transcription start site (TSS) and +1Kb of the transcription termination site (TTS) of rhythmic genes differentially regulated in Clk knockouts (right) in all genotypes at ZT04 and at ZT16. (TIF) [file pgen.1008265.s015.tif]
